# Correlation between serum iron levels and pulmonary function: A cross-sectional analysis based on NHANES database 5319 cases

**DOI:** 10.1097/MD.0000000000036449

**Published:** 2023-12-15

**Authors:** Lang Su, Sheng Hu, Silin Wang, Qiang Guo, Yiping Wei

**Affiliations:** a Department of Thoracic Surgery, The Second Affiliated Hospital of Nanchang University, Nanchang, China.

**Keywords:** FEV1, FVC, NHANES, pulmonary function, serum iron

## Abstract

Pulmonary function, one of the main indicators of respiratory system assessment, is difficult to measure in specific cases. The study investigated the association between serum iron levels and pulmonary function. The cross-sectional study was conducted using data from 5319 participants from the 2010–2012 National Health and Nutrition Examination Survey. Forced expiratory volume in 1 second (FEV1), forced vital capacity (FVC), and forced expiratory flow from 25% to 75% of FVC were used as indicators of pulmonary function to analyze the relationship of serum iron and pulmonary function. Univariate and stratified analyses, multiple equation regression analysis, smoothed curve fitting analysis, and threshold effect analysis were performed to explore the relationship between pulmonary function and serum iron concentrations. Threshold effect analysis revealed a nonlinear relationship between serum iron levels and FVC, as well as FEV1, with inflection points observed at 8.1 (µmol/L) and 8.4 (µmol/L), respectively. When serum iron concentrations fell below the inflection point, there was no statistically significant relationship between serum iron and FVC (*P* = .065) or FEV1 (*P* = .095) (*P* > .005). However, when serum iron concentrations exceeded the inflection point, both FVC (β = 6.87; 95% confidence interval [CI] = 3.95, 9.79; *P* < .0001) and FEV1 (β = 7.09; 95% CI = 4.54, 9.64; *P* < .0001) exhibited a positive correlation with increasing serum iron levels. Additionally, forced expiratory flow from 25% to 75% of FVC (mL/s) demonstrated a positive association with serum iron (β = 6.72; 95% CI = 2.30, 11.13; *P* = .0029). Serum iron level was positively correlated with pulmonary function within a certain range of serum iron concentration. Serum iron level may be a protective factor for pulmonary function.

## 1. Introduction

Pulmonary function is one of the key indicators for detecting respiratory diseases and is an important indicator for the functions of pulmonary ventilation and pulmonary defense.^[1]^ There are many clinical indicators used for pulmonary function assessment, such as vital capacity, forced expiratory volume in 1 second (FEV1), forced vital capacity (FVC), FEV1/FVC, forced expiratory flow from 25% to 75% of FVC (FEF 25%–75%), and general blood gas.^[1–3]^ However, in some populations, for example, coma, shock, mental disorders, and other patients who cannot cooperate, the above indicators can be difficult to obtain. Additionally, testing pulmonary function requires cooperation between the examiner and the patient, and the results can be influenced by a variety of technical and personal factors.^[4]^ Blood gas analysis is an acute indicator that can only reflect the immediate situation of the patient. Blood gas analysis is not practical for assessing the overall pulmonary function of the patient.^[5]^

Serum iron is a trace element in the human body, which plays an important role in numerous physiological and pathological mechanisms of the human body.^[6,7]^ Some studies have shown that serum iron is positively correlated with the cardiovascular system and renal function,^[8,9]^ McKeever et al^[10]^ demonstrated that higher serum iron was independently associated with higher FEV1.However, the association between serum iron and pulmonary function is uncertain. The National Health and Nutrition Examination Survey (NHANES) is a cross-sectional population-based database that annually surveys a nationally representative sample of people located in every state in the United States.^[11]^ It includes diet- and health-related questions, demographic data, general screening data, laboratory data, etc. These data are used to determine disease risk factors and disease prevalence.^[12,13]^ The wide sample of the database provides more reliable support for study. The aim of this study was to investigate whether serum iron levels affect lung function. The data used in this article are extracted from the NHANES database. The purpose of this study is to explore new predictors for the assessment of pulmonary function.

## 2. Materials and methods

### 2.1. Study population

Data were downloaded by logging into the NHANES database website (Centers for Disease Control and Prevention, https://www.cdc.gov/), selecting the data from the NHANES database for 2010–2012, then selecting the laboratory data option and study variable data, exporting the data in XPT format, and using R Studio for viewing. Retrospective analysis was then performed using R software and EmpowerStats(www.empowerstats.com). Data exclusion criteria included missing FVC, FEF 25%–75%, and FEV1 data, missing serum iron data, participants who were already pregnant and those missing from thoracoabdominal surgery, and individuals with stroke, heart attack, and cigarette date.

### 2.2. Variables

The exposure variables in this study were serum iron levels (µmol/L) and the outcome variable pulmonary function, with FVC, FEV1, and FEF 25%–75% selected for pulmonary function as the outcome assessment index. Three outcome variables were divided into 3 different groups and analyzed by R software and EmpowerStats. Subgroups were based on serum iron concentration tertile as low, medium, and high. Fourteen covariates were added for more accurate study results, including age (years), gender, race/Hispanic origin, education level, thoracic/abdominal surgery, respiratory disease, standing height (cm) tertile, body mass index (kg/m^2^), systolic blood pressure (mm Hg), diastolic blood pressure (mm Hg), alcohol, had food, diabetes, and cholesterol (mmol/L) tertile (Table 1). R software and EmpowerStats were used to analyze the relationship between changes in serum iron concentration and pulmonary function. The results were analyzed by observing β and *P* values. A standardized β value >0 is considered to be a coefficient of increase in pulmonary function and <0 is considered to be a coefficient of decay in pulmonary function.

**Table 1:** Baseline characteristics of participants (N = 5319).

Iron, refrigerated (µmol/L) tertile	Mean + SD	Low	Medium	High	*P* value*
N		1751	1783	1785	
Baseline FVC (mL)	3964.29 ± 1051.14	3688.53 ± 979.74	3967.04 ± 1061.25	4232.04 ± 1039.55	<.001
Baseline FEV1 (mL)	3172.44 ± 879.68	2963.91 ± 831.04	3154.93 ± 884.72	3394.49 ± 868.97	<.001
Baseline FEF 25%–75% (mL/s)	3145.77 ± 1290.35	2999.60 ± 1251.48	3091.69 ± 1307.16	3343.16 ± 1287.36	<.001
Iron, refrigerated (µmol/L)	15.48 ± 6.37	9.07 ± 2.38	14.77 ± 1.49	22.48 ± 4.82	<.001
Age (years)	39.88 ± 18.98	39.97 ± 19.19	41.36 ± 19.16	38.31 ± 18.48	<.001
Standing height (cm)	167.45 ± 10.13	165.31 ± 10.01	167.79 ± 10.12	169.20 ± 9.89	<.001
Body mass index (kg/m^2^)	28.14 ± 6.91	29.78 ± 7.89	28.11 ± 6.57	26.57 ± 5.73	<.001
Systolic blood pressure (mm Hg)	119.66 ± 17.35	119.96 ± 17.28	120.55 ± 18.22	118.50 ± 16.47	.002
Diastolic blood pressure (mm Hg)	68.30 ± 13.22	67.82 ± 13.44	68.83 ± 13.14	68.25 ± 13.06	.092
Cholesterol (mmol/L)	4.86 ± 1.07	4.73 ± 1.02	4.91 ± 1.07	4.93 ± 1.11	<.001
N (%)					
Age (years)					<.001
<40	2688 (50.54%)	871 (49.74%)	855 (47.95%)	962 (53.89%)	
≥40, <60	1557 (29.27%)	514 (29.35%)	525 (29.44%)	518 (29.02%)	
≥60	1074 (20.19%)	366 (20.90%)	403 (22.60%)	305 (17.09%)	
Gender					<.001
Male	2668 (50.16%)	623 (35.58%)	939 (52.66%)	1106 (61.96%)	
Female	2651 (49.84%)	1128 (64.42%)	844 (47.34%)	679 (38.04%)	
Race/Hispanic origin					<.001
Mexican American	1146 (21.55%)	356 (20.33%)	380 (21.31%)	410 (22.97%)	
Other Hispanic	608 (11.43%)	200 (11.42%)	211 (11.83%)	197 (11.04%)	
Non-Hispanic White	2321 (43.64%)	688 (39.29%)	792 (44.42%)	841 (47.11%)	
Non-Hispanic Black	954 (17.94%)	429 (24.50%)	312 (17.50%)	213 (11.93%)	
Other races, including multiracial	290 (5.45%)	78 (4.45%)	88 (4.94%)	124 (6.95%)	
Education level					.063
<9th grade	467 (8.78%)	151 (8.62%)	168 (9.42%)	148 (8.29%)	
9th–11th grade	633 (11.90%)	208 (11.88%)	230 (12.90%)	195 (10.92%)	
High school graduate	957 (17.99%)	313 (17.88%)	330 (18.51%)	314 (17.59%)	
Some college or AA degree	1231 (23.14%)	427 (24.39%)	398 (22.32%)	406 (22.75%)	
College graduate or above	932 (17.52%)	272 (15.53%)	323 (18.12%)	337 (18.88%)	
Unknown	1099 (20.66%)	380 (21.70%)	334 (18.73%)	385 (21.57%)	
Thoracic/abdominal surgery					<.001
Yes	933 (17.54%)	343 (19.59%)	332 (18.62%)	258 (14.45%)	
No	4386 (82.46%)	1408 (80.41%)	1451 (81.38%)	1527 (85.55%)	
Respiratory disease					<.001
Yes	1048 (19.70%)	386 (22.04%)	332 (18.62%)	330 (18.49%)	
No	4118 (77.42%)	1297 (74.07%)	1398 (78.41%)	1423 (79.72%)	
Unknown	153 (2.88%)	68 (3.88%)	53 (2.97%)	32 (1.79%)	
Standing height (cm) tertile					<.001
Low	1773 (33.33%)	732 (41.80%)	559 (31.35%)	482 (27.00%)	
Medium	1772 (33.31%)	577 (32.95%)	613 (34.38%)	582 (32.61%)	
High	1774 (33.35%)	442 (25.24%)	611 (34.27%)	721 (40.39%)	
Body mass index (kg/m^2^)					<.001
<25	1897 (35.66%)	521 (29.75%)	610 (34.21%)	766 (42.91%)	
≥25	3422 (64.34%)	1230 (70.25%)	1173 (65.79%)	1019 (57.09%)	
Systolic blood pressure (mm Hg)					.005
<140	4396 (82.65%)	1429 (81.61%)	1445 (81.04%)	1522 (85.27%)	
≥140	601 (11.30%)	203 (11.59%)	218 (12.23%)	180 (10.08%)	
Unknown	322 (6.05%)	119 (6.80%)	120 (6.73%)	83 (4.65%)	
Diastolic blood pressure (mm Hg)					.036
<90	4745 (89.21%)	1553 (88.69%)	1572 (88.17%)	1620 (90.76%)	
≥90	252 (4.74%)	79 (4.51%)	91 (5.10%)	82 (4.59%)	
Unknown	322 (6.05%)	119 (6.80%)	120 (6.73%)	83 (4.65%)	
Alcohol					<.001
Yes	2970 (55.84%)	878 (50.14%)	1009 (56.59%)	1083 (60.67%)	
No	956 (17.97%)	394 (22.50%)	334 (18.73%)	228 (12.77%)	
Unknown	1393 (26.19%)	479 (27.36%)	440 (24.68%)	474 (26.55%)	
Had food					.130
Yes	1273 (23.93%)	424 (24.21%)	412 (23.11%)	437 (24.48%)	
No	3910 (73.51%)	1275 (72.82%)	1319 (73.98%)	1316 (73.73%)	
Unknown	136 (2.56%)	52 (2.97%)	52 (2.92%)	32 (1.79%)	
Diabetes					.009
Yes	383 (7.20%)	156 (8.91%)	124 (6.95%)	103 (5.77%)	
No	4853 (91.24%)	1568 (89.55%)	1629 (91.36%)	1656 (92.77%)	
Unknown	83 (1.56%)	27 (1.54%)	30 (1.68%)	26 (1.46%)	
Cholesterol (mmol/L) tertile					<.001
Low	1726 (32.45%)	641 (36.61%)	540 (30.29%)	545 (30.53%)	
Medium	1790 (33.65%)	607 (34.67%)	605 (33.93%)	578 (32.38%)	
High	1803 (33.90%)	503 (28.73%)	638 (35.78%)	662 (37.09%)	

Mean + SD/N(%), outcome variables: baseline FVC (mL), baseline FEV1 (mL), baseline FEF 25%–75% (mL/s); exposure variable: serum iron(µmol/L). This table was generated using EasyTok statistical software (www.empowerstats.com) and R software.

FEF 25%–75% = forced expiratory flow from 25% to 75% of FVC, FEV1 = forced expiratory volume in 1 second, FVC = forced vital capacity, SD = standard deviation.

* *P* value: Kruskal-Wallis rank sum test for continuous variables, Fisher exact probability test for count variables with theoretical number < 10.

### 2.3. Statistical analysis

**Figure 1.**
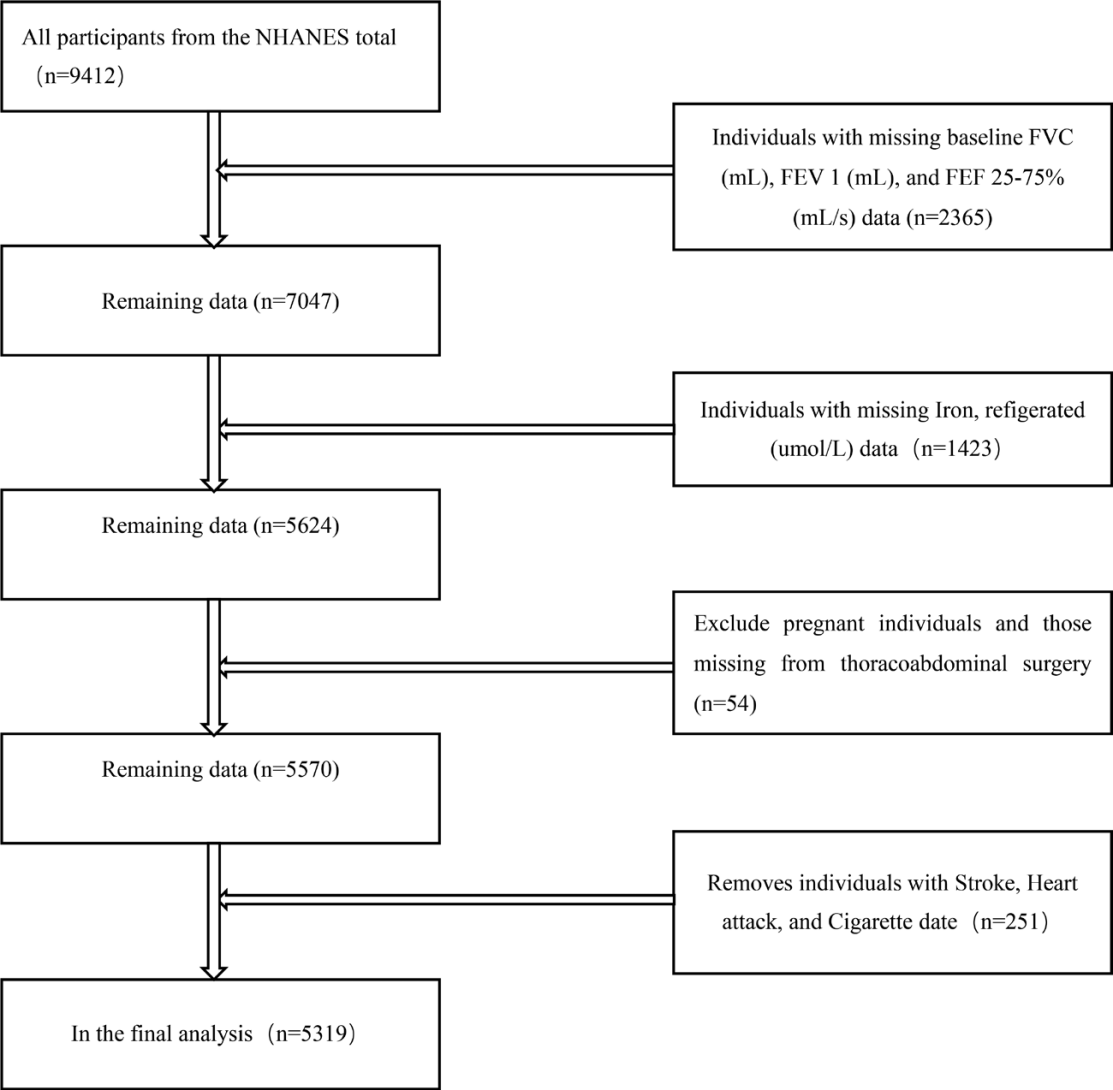
Participants’ screening flowchart.

The results were generated by Empower Statistics and R software. Univariate and multiple regression analyses were performed on the 3 serum iron concentration groups. Two model adjustments in the subgroup analyses were performed. The first adjustment variable included age, gender, and race and the second adjustment variables included age (years), gender, race/Hispanic origin, education level, thoracic/abdominal surgery, respiratory disease, standing height (cm) tertile, body mass index (kg/m^2^), systolic blood pressure (mm Hg), diastolic blood pressure (mm Hg), alcohol, had food, diabetes, and cholesterol (mmol/L) tertile (Table 3). Smoothed fitting curves were used to analyze the association between exposure variable and dependent variable (Fig. 1).

**Table 2:** Univariate analysis of serum iron and pulmonary function.

Statistics	Statistics	Baseline FVC (mL)	Baseline FEV1 (mL)	Baseline FEF 25%–75% (mL/s)
	N	β (95% CI) *P*	β (95% CI) *P*	β (95% CI) *P*
Iron, refrigerated (µmol/L)	15.48 ± 6.37	35.19 (30.85, 39.53) < .0001	27.87 (24.23, 31.51) < .0001	22.21 (16.79, 27.62) < .0001
Iron, refrigerated(µmol/L) tertile				
Low	1751 (32.92%)	Reference	Reference	Reference
Medium	1783 (33.52%)	278.51 (210.74, 346.29) < .0001	191.02 (134.17, 247.86) < .0001	92.09 (7.53, 176.66) .0328
High	1785 (33.56%)	543.52 (475.77, 611.27) < .0001	430.57 (373.74, 487.41) < .0001	343.56 (259.02, 428.11) < .0001
Age (years)	39.88 ± 18.98	−16.26 (−17.68, −14.83) < .0001	−21.60 (−22.70, −20.50) < .0001	−38.13 (−39.64, −36.62) < .0001
Age (years)				
<40	2688 (50.54%)	Reference	Reference	Reference
≥40, <60	1557 (29.27%)	−302.44 (−363.86, −241.03) < .0001	−475.79 (−523.53, −428.05) < .0001	−896.92 (−963.96, −829.88) < .0001
≥60	1074 (20.19%)	−974.49 (−1044.10, −904.87) < .0001	−1130.05 (−1184.16, −1075.94) < .0001	−1829.52 (−1905.51, −1753.53) < .0001
Gender				
Male	2668 (50.16%)	Reference	Reference	Reference
Female	2651 (49.84%)	−1245.54 (−1291.06, −1200.03) < .0001	−908.11 (−948.61, −867.61) < .0001	−640.03 (−707.22, −572.83) < .0001
Race/Hispanic origin				
Mexican American	1146 (21.55%)	Reference	Reference	Reference
Other Hispanic	608 (11.43%)	−159.20 (−259.67, −58.74) .0019	−153.03 (−238.11, −67.95) .0004	−196.52 (−322.34, −70.70) .0022
Non-Hispanic White	2321 (43.64%)	249.12 (176.83, 321.41) < .0001	43.73 (−17.49, 104.95) .1615	−374.43 (−464.97, −283.90) < .0001
Non-Hispanic Black	954 (17.94%)	−414.57 (−502.32, −326.81) < .0001	−389.07 (−463.39, −314.75) < .0001	−472.29 (−582.20, −362.39) < .0001
Other races, including multiracial	290 (5.45%)	−154.97 (−286.58, −23.35) .0211	−124.74 (−236.20, −13.27) .0283	−141.99 (−306.83, 22.85) .0914
Education level				
<9th grade	467 (8.78%)	Reference	Reference	Reference
9th–11th grade	633 (11.90%)	248.98 (124.58, 373.39) < .0001	198.57 (95.70, 301.44) .0002	154.89 (7.16, 302.61) .0399
High school graduate	957 (17.99%)	377.93 (262.81, 493.05) < .0001	276.47 (181.27, 371.66) < .0001	154.44 (17.74, 291.13) .0268
Some college or AA degree	1231 (23.14%)	376.80 (265.96, 487.64) < .0001	321.95 (230.30, 413.61) < .0001	279.47 (147.86, 411.08) < .0001
College graduate or above	932 (17.52%)	596.97 (481.35, 712.60) < .0001	478.45 (382.84, 574.06) < .0001	375.59 (238.30, 512.89) < .0001
Unknown	1099 (20.66%)	416.62 (303.97, 529.27) < .0001	635.54 (542.39, 728.70) < .0001	1110.82 (977.05, 1244.58) < .0001
Thoracic/abdominal surgery				
Yes	933 (17.54%)	Reference	Reference	Reference
No	4386 (82.46%)	492.09 (418.99, 565.18) < .0001	518.34 (457.75, 578.93) < .0001	776.42 (687.65, 865.19) < .0001
Respiratory disease				
Yes	1048 (19.70%)	Reference	Reference	Reference
No	4118 (77.42%)	141.56 (71.12, 212.00) < .0001	128.09 (69.14, 187.05) < .0001	136.71 (49.59, 223.82) .0021
Unknown	153 (2.88%)	−797.73 (−973.93, −621.53) < .0001	−647.30 (−794.78, −499.83) < .0001	−554.89 (−772.81, −336.97) < .0001
Standing height (cm)	167.45 ± 10.13	75.14 (73.22, 77.06) < .0001	54.95 (53.14, 56.76) < .0001	37.89 (34.62, 41.15) < .0001
Standing height (cm) tertile				
Low	1773 (33.33%)	Reference	Reference	Reference
Medium	1772 (33.31%)	743.97 (692.60, 795.35) < .0001	555.89 (508.92, 602.86) < .0001	371.49 (289.76, 453.21) < .0001
High	1774 (33.35%)	1719.87 (1668.51, 1771.23) < .0001	1257.99 (1211.03, 1304.94) < .0001	862.41 (780.71, 944.11) < .0001
Body mass index (kg/m^2^)	28.14 ± 6.91	−12.70 (−16.77, −8.62) < .0001	−14.95 (−18.35, −11.55) < .0001	−17.06 (−22.06, −12.06) < .0001
Body mass index (kg/m^2^)				
<25	1897 (35.66%)	Reference	Reference	Reference
≥25	3422 (64.34%)	−59.20 (−118.16, −0.24) .0491	−154.83 (−204.01, −105.65) < .0001	−280.85 (−352.86, −208.84) < .0001
Systolic blood pressure (mm Hg)	119.66 ± 17.35	−5.72 (−7.40, −4.05) < .0001	−9.47 (−10.85, −8.09) < .0001	−18.66 (−20.65, −16.67) < .0001
Systolic blood pressure (mm Hg)				
<140	4396 (82.65%)	Reference	Reference	Reference
≥140	601 (11.30%)	−552.24 (−640.47, −464.00) < .0001	−619.62 (−692.68, −546.56) < .0001	−938.59 (−1045.67, −831.52) < .0001
Unknown	322 (6.05%)	−313.22 (−430.36, −196.09) < .0001	−227.29 (−324.28, −130.31) < .0001	−140.41 (−282.55, 1.73) .0529
Diastolic blood pressure (mm Hg)	68.30 ± 13.22	6.46 (4.26, 8.66) < .0001	0.33 (−1.51, 2.17) .7254	−7.31 (−10.01, −4.62) < .0001
Diastolic blood pressure (mm Hg)				
<90	4745 (89.21%)	Reference	Reference	Reference
≥90	252 (4.74%)	60.23 (−72.76, 193.22) .3747	−62.37 (−173.74, 49.00) .2724	−200.84 (−364.27, −37.41) .0160
Unknown	322 (6.05%)	−243.77 (−362.24, −125.30) < .0001	−155.92 (−255.13, −56.70) .0021	−37.65 (−183.24, 107.94) .6122
Alcohol				
Yes	2970 (55.84%)	Reference	Reference	Reference
No	956 (17.97%)	−700.54 (−774.81, −626.27) < .0001	−495.78 (−557.99, −433.58) < .0001	−281.45 (−372.46, −190.44) <.0001
Unknown	1393 (26.19%)	−163.23 (−228.09, −98.36) < .0001	131.28 (76.96, 185.60) < .0001	634.33 (554.85, 713.81) < .0001
Had food				
Yes	1273 (23.93%)	Reference	Reference	Reference
No	3910 (73.51%)	−95.81 (−162.22, −29.39) .0047	−96.13 (−151.71, −40.55) .0007	−117.57 (−199.13, −36.01) .0047
Unknown	136 (2.56%)	−272.59 (−458.26, −86.92) .0040	−159.96 (−315.35, −4.58) .0437	5.57 (−222.45, 233.58) .9618
Diabetes				
Yes	383 (7.20%)	Reference	Reference	Reference
No	4853 (91.24%)	667.68 (559.86, 775.50) < .0001	634.64 (544.80, 724.48) < .0001	773.96 (641.44, 906.49) < .0001
Unknown	83 (1.56%)	321.34 (75.39, 567.30) .0105	289.37 (84.43, 494.32) .0057	298.97 (−3.35, 601.28) .0526
Cholesterol (mmol/L)	4.86 ± 1.07	−56.05 (−82.39, −29.70) < .0001	−107.72 (−129.61, −85.83) < .0001	−221.07 (−252.9, −189.23) < .0001
Cholesterol (mmol/L) tertile				
Low	1726 (32.45%)	Reference	Reference	Reference
Medium	1790 (33.65%)	−45.38 (−114.84, 24.08) .2004	−127.99 (−185.78, −70.21) < .0001	−285.35 (−369.41, −201.30) <.0001
High	1803 (33.90%)	−105.04 (−174.37, −35.70) .0030	−248.62 (−306.31, −190.94) < .0001	−546.97 (−630.88, −463.07) <.0001

Statistics: number of people as a percentage, β, CI, *P*. Exposure variable: Iron, refrigerated (µmol/L), Iron, refrigerated (µmol/L) tertile, age (years), gender, race/Hispanic origin, education level, thoracic/abdominal surgery, respiratory disease, standing height (cm), body mass index (kg/m^2^), systolic blood pressure (mm Hg), systolic blood pressure (mm Hg), diastolic blood pressure (mm Hg), diastolic blood pressure (mm Hg), alcohol, had food, diabetes, cholesterol (mmol/L). This table was generated using EasyTok statistical software (www.empowerstats.com) and R software.

β = beta value, CI = confidence interval, FEF 25%–75% = forced expiratory flow from 25% to 75% of FVC, FEV1 = forced expiratory volume in 1 second, FVC = forced vital capacity, *P* = *P* value.

**Table 3:** Multiple regression equation analysis of all covariate.

Exposure	Nonadjusted	Adjust I	Adjust II
	β (95% CI) *P*	β (95% CI) *P*	β (95% CI) *P*
Baseline FVC (mL)			
Iron, refrigerated (µmol/L)	35.19 (30.85, 39.53) < .0001	7.52 (4.40, 10.64) < .0001	5.45 (2.78, 8.13) < .0001
Iron, refrigerated (µmol/L) tertile			
Low	Reference	Reference	Reference
Medium	278.51 (210.74, 346.29) < .0001	45.43 (−2.30, 93.16) .0622	20.50 (−19.94, 60.94) .3204
High	543.52 (475.77, 611.27) < .0001	115.07 (66.31, 163.82) < .0001	79.32 (37.65, 120.99) .0002
Baseline FEV1 (mL)			
Iron, refrigerated (µmol/L)	27.87 (24.23, 31.51) < .0001	7.07 (4.48, 9.66) < .0001	5.80 (3.49, 8.11) < .0001
Iron, refrigerated (µmol/L) tertile			
Low	Reference	Reference	Reference
Medium	191.02 (134.17, 247.86) < .0001	30.63 (−8.97, 70.23) .1295	16.22 (−18.77, 51.21) .3636
High	430.57 (373.74, 487.41) < .0001	104.55 (64.10, 145.00) < .0001	82.40 (46.34, 118.45) < .0001
Baseline FEF 25%–75% (mL/s)			
Iron, refrigerated (µmol/L)	22.21 (16.79, 27.62) < .0001	6.39 (1.92, 10.85) .0051	6.72 (2.30, 11.13) .0029
Iron, refrigerated (µmol/L) tertile			
Low	Reference	Reference	Reference
Medium	92.09 (7.53, 176.66) .0328	5.63 (−62.66, 73.93) .8715	8.10 (−58.71, 74.92) .8121
High	343.56 (259.02, 428.11) < .0001	86.07 (16.31, 155.84) .0156	89.96 (21.12, 158.81) .0105

β (95% CI) *P* value. Exposure variable: serum iron (µmol/L). Nonadjusted model adjust for: none. Adjust I model adjust for: age, gender, race/Hispanic origin. Adjust II model adjust for: age, gender, race/Hispanic origin, education level, thoracic/abdominal surgery, respiratory disease, cigarette, heart attack, stroke, body mass index, systolic blood pressure, diastolic blood pressure, standing height (cm). Outcome variables: baseline FVC (mL); baseline FEV1 (mL), baseline FEF 25%–75% (mL/s). This table was generated using EasyTok statistical software (www.empowerstats.com) and R software.

β = beta value, CI = confidence interval, FEF 25%–75% = forced expiratory flow from 25% to 75% of FVC, FEV1 = forced expiratory volume in 1 second, FVC = forced vital capacity, *P* = *P* value.

## 3. Results

### 3.1. Selected participants’ baseline characteristics

After the initial screening, data were obtained for 9412 participants. A total of 2365 participants with missing FVC, FEF 25%–75%, and FEV1 data, 1423 participants with missing serum iron data, and 54 participants who were already pregnant and those missing from thoracoabdominal surgery, and 251 participants with stroke, heart attack, and cigarette date were all excluded, leaving 5319 participants included in the study, in which 2668 were males and 2651 were females with a mean age of 39.88 and a standard deviation of 18.98 (Fig. 1 and Table 1).

Subgroups were categorized by serum iron concentrations (low concentration group: 1.10–12.20µmol/L, medium concentration group: 12.40–17.40 µmol/L, and high concentration group: 17.60–61.40 µmol/L). The mean values of the tertile were 9.07, 14.77, and 22.48 µmol/L. Table 1 shows the baseline table. The grouped data were derived using Kruskal-Wallis rank sum test. N = 1751 for the low concentration group, N = 1783 for the medium concentration group, and N = 1785 for the high concentration group, with no significant difference in the number of participants among the 3 groups (*P <* .0001). The average heights of the 3 groups were 156.41 cm, 167.04 cm, and 178.88 cm, respectively. The mean values of serum cholesterol in the 3 groups were 3.74 mmol/L, 4.74 mmol/L, and 6.04 mmol/L, respectively (*P* < .001). The results indicate that, among the covariates, age (years), gender, race/Hispanic origin, thoracic/abdominal surgery, respiratory disease, standing height (cm) tertile, body mass index (kg/m^2^), systolic blood pressure (mm Hg), alcohol consumption, having food, diabetes, and cholesterol (mmol/L) tertile were all statistically significant (*P* < .05). In contrast, education level and diastolic blood pressure did not show statistical significance (*P* > .05).

### 3.2. Analysis of the association between serum iron and pulmonary function on a univariate basis

Serum iron was positively correlated with pulmonary function. Univariate analysis showed that FVC, FEV1, and FEF 25%–75% were all *P* < .0001. In the univariate analysis, age (years), gender, race/Hispanic origin, education level, thoracic/abdominal surgery, respiratory disease, standing height (cm) tertile, body mass index (kg/m^2^), systolic blood pressure (mm Hg), alcohol, had food, diabetes, and cholesterol (mmol/L) tertile were statistically significant (*P* < .05). On the other hand, diastolic blood pressure did not exhibit statistical significance (*P* > .05). The results revealed that individuals over the age of 60 faced a higher risk compared with those aged 40 to 60, and serum iron concentrations were predominantly higher in men (Table 2). The β value increased with higher education levels (*P* < .05). Risk factors were elevated with higher body mass index and serum cholesterol levels. The β values for the group without diabetes, no history of thoracic and abdominal surgery, and no respiratory disease were significantly higher than that of the control group (*P* < .0001). Furthermore, patients with no history of alcohol consumption exhibited higher risk factors (*P* < .0001). Serum iron exhibited a positive correlation with lung function. The confidence intervals (CIs) for *P* values of different covariates are presented in Table 2.

### 3.3. Serum iron and pulmonary function analyzed by multiple regression equations

Further analysis of the association between serum iron and pulmonary function by multiple regression revealed that serum iron was positively associated with FVC, FEV1, and FEF 25%–75%. Two adjustments were performed for covariates after grouping serum iron concentrations. In the high serum iron concentration group, the *P* values for FVC, FEV1, and FEF 25%–75% were statistically significant both before and after variable adjustment. Moreover, with the low concentration group as the reference baseline, the high concentration group showed significantly higher β values for all 3 indexes compared with the medium concentration group. The β values increased with the increase of iron level. The β values, CIs, and *P* values of all covariates before and after adjustment are shown in Table 3. Table S1, Supplemental Digital Content, http://links.lww.com/MD/L24 shows multiple regression equation analysis for all subgroup variables (Table S1, Supplemental Digital Content, http://links.lww.com/MD/L24, the multiple regression analysis of all subgroup variables).

### 3.4. Statistical analysis of smooth curve fitting, threshold effect, and saturation effect between serum iron levels and pulmonary function

**Figure 2.**
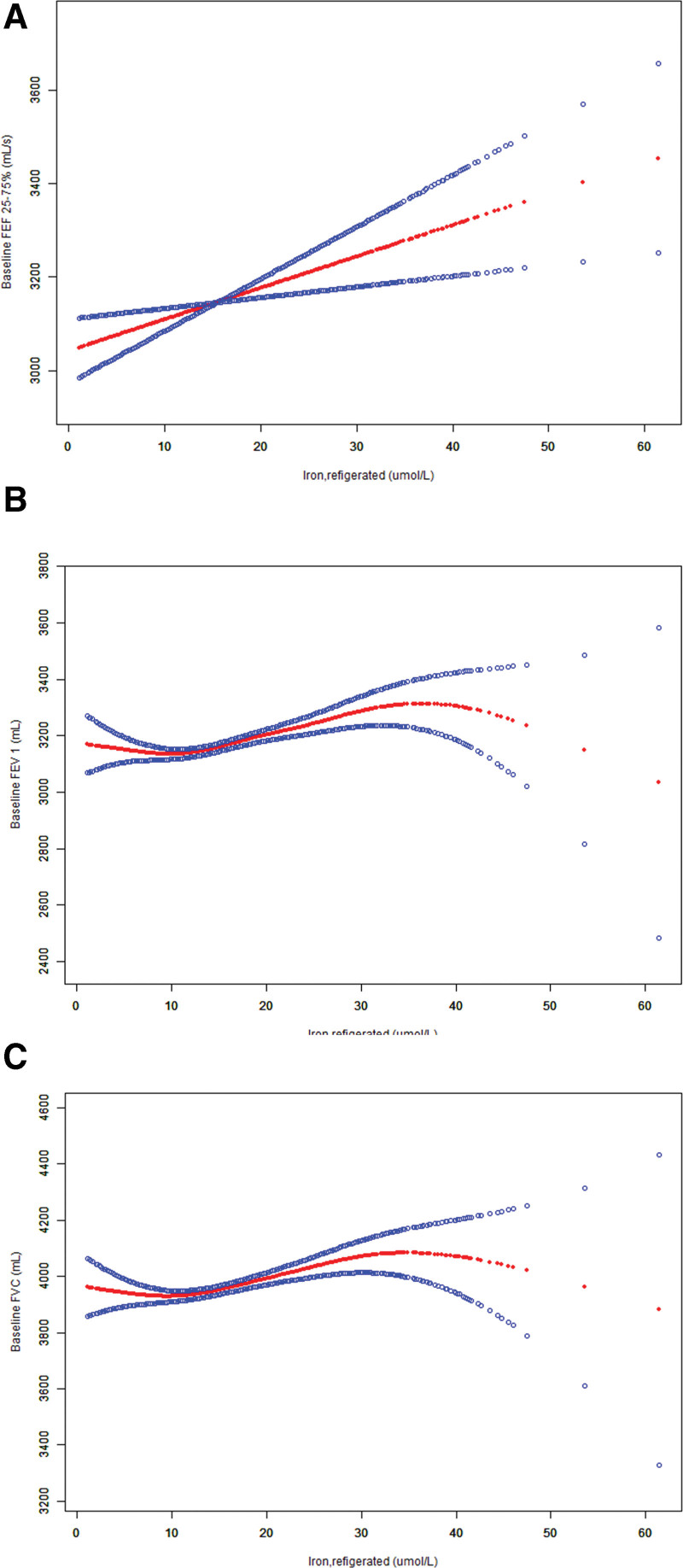
The association between serum iron and pulmonary function. The smooth curve fit between the variables is indicated by the red line. The fit’s 95% confidence interval is represented by the blue bar. (A)Scatter plot of curve fitting of serum iron levels and FEF 25%–75%. (B) Scatter plot of curve fitting of serum iron levels and FEV1. (C) Scatter plot of curve fitting of serum iron levels and FVC. Adjusted for age(years), gender, race/Hispanic origin, education level, thoracic/abdominal surgery, respiratory disease, standing height (cm) tertile, body mass index (kg/m^2^), systolic blood pressure (mm Hg), diastolic blood pressure (mm Hg), alcohol, had food, diabetes, cholesterol (mmol/L) tertile. FEF 25%–75% = forced expiratory flow from 25% to 75% of FVC, FEV1 = forced expiratory volume in 1 second, FVC = forced vital capacity.

Threshold effect analysis revealed a nonlinear relationship between serum iron levels and FVC, as well as FEV1, with inflection points observed at 8.1 (µmol/L) and 8.4 (µmol/L), respectively. When serum iron concentrations fell below the inflection point, there was no statistically significant relationship between serum iron and FVC (*P* = .065) or FEV1 (*P* = .095) (*P* > .005). However, when serum iron concentrations exceeded the inflection point, both FVC (β = 6.87; 95% CI = 3.95, 9.79; *P* < .0001) and FEV1 (β = 7.09; 95% CI = 4.54, 9.64; *P* < .0001) exhibited a positive correlation with increasing serum iron levels. Additionally, FEF 25%–75% (mL/s) demonstrated a positive association with serum iron (β = 6.72; 95% CI = 2.30, 11.13; *P* = .002). The covariates used for adjustment are detailed in Table 4. Smoothing curve fitting was used to assess whether serum iron levels were related to pulmonary function. The results indicated that serum iron exhibited a nonlinear correlation with FEV1 and FVC. However, there was a linear correlation between serum iron and FEF 25%–75%. Beyond the inflection point, FEV1 and FVC exhibited a significant increase with rising serum iron concentration until reaching a plateau. FEF 25%–75% displayed a linear positive correlation with increasing serum iron concentration (Fig. 2). In the smooth curve analysis depicting the relationship between serum iron and FVC, most covariables demonstrated that FVC increased as serum iron concentration increased. The curve fitting for individuals with body mass index >25 and diastolic blood pressure > 90 exhibited substantial fluctuations, demonstrating an S-shaped curve (Figure S1, Supplemental Digital Content, http://links.lww.com/MD/L25, the smooth fitting curves of all covariables with FVC). In the smoothed curve analysis of the relationship between serum iron and FEV1, most of the covariates showed a concomitant increase in FEV1 with increasing serum iron. The curve fitting for individuals with diastolic blood pressure > 90 exhibited substantial fluctuations, demonstrating an S-shaped curve (Figure S2, Supplemental Digital Content, http://links.lww.com/MD/L26, the smooth fitting curves of all covariables with FEV1). In a smoothed curve analysis of the relationship between serum iron and FEF 25%–75%. The curve fitting for all covariables displayed an upward trend (Figure S3, Supplemental Digital Content, http://links.lww.com/MD/L27, the smooth fitting curves of all covariables with FEF 25%–75%).

**Table 4:** Analysis of threshold effect.

Outcome:	Baseline FVC (mL)	Baseline FEV1 (mL)	Baseline FEF 25%–75% (mL/s)
Model I			
A straight line effect	5.45 (2.78, 8.13) < .0001	5.80 (3.49, 8.11) < .0001	6.72 (2.30, 11.13) .0029
Model II			
Break point (k)	8.1	8.4	13.3
<k-segment effect 1	−20.56 (−42.42, 1.31) .0655	−14.85 (−32.29, 2.59) .0952	−1.68 (−14.37, 11.01) .7954
>k-segment effect 2	6.87 (3.95, 9.79) < .0001	7.09 (4.54, 9.64) < .0001	9.69 (3.59, 15.79) .0019
The effect difference between 2 and 1	27.43 (4.54, 50.31) .0189	21.94 (3.57, 40.30) .0193	11.37 (−4.74, 27.48) .1667
Predicted value of equation at break point	3709.71 (3661.58, 3757.84)	2966.77 (2926.78, 3006.75)	3050.48 (2995.38, 3105.58)
Log likelihood ratio tests	.019	.019	.165

β (95% CI) *P* value. Exposure variable: serum iron(µmol/L). Adjustment variables: age (years), gender; race/Hispanic origin, education level; thoracic/abdominal surgery, respiratory disease, standing height (cm) tertile, body mass index (kg/m^2^), systolic blood pressure (mm Hg), diastolic blood pressure (mm Hg), alcohol, had food, diabetes, cholesterol (mmol/L) tertile. Outcome variables: baseline FVC (mL); baseline FEV1 (mL), baseline FEF 25%–75% (mL/s). This table was generated using EasyTok statistical software (www.empowerstats.com) and R software.

β = beta value, CI = confidence interval, FEF 25%–75% = forced expiratory flow from 25% to 75% of FVC, FEV1 = forced expiratory volume in 1 second, FVC = forced vital capacity, *P* = *P* value.

## 4. Discussion

Respiratory diseases are becoming increasingly prominent in today’s society, affecting the quality of survival and life expectancy of patients.^[14–20]^ Pulmonary function, as the main assessment indicator of the respiratory system, plays a crucial role in the diagnosis and treatment of diseases; however, there are some contraindications to pulmonary function tests.^[21,22]^ Currently, the limitations of pulmonary function tests have not been addressed by the introduction of better tests.^[21]^ This study found that serum iron concentrations may be a good indicator for assessing pulmonary function.

The regulation between pulmonary function and serum iron concentrations was analyzed by screening the NHANES database, a population-based cross-sectional survey that surveys a nationally representative sample each year. This study obtained 5319 participants eligible for our study.^[11]^ Many researchers have found many meaningful studies through this database. The study yielded interesting and noteworthy findings from the analysis of large, rigorous, and authentic data from the NHANES database.

Serum iron, an important element in the body, plays a crucial role in maintaining homeostatic balance in the body. Serum iron is essential for all cell division and is required for DNA biosynthesis. Iron is required for daily metabolism and biosynthesis, mainly for the biosynthesis of hemoglobin to meet the daily requirement of red blood cells.^[23]^ Serum iron is involved in the synthesis of many proteins and plays an important role in proteins related to oxygen metabolism. The oxygen-carrying capacity of hemoglobin directly affects the pulmonary function.^[24]^ Iron deficiency has been shown to be associated with reduced exercise capacity, peak oxygen consumption, and 6-minute walk test values in humans. It is also associated with poor prognosis and quality of life in patients with chronic heart failure.^[25–27]^ New study by Ueda et al^[8]^ showed that serum iron concentration can be a predictor of adverse outcomes in patients with acute decompensated heart failure. Del Greco et al^[28]^ study found positive effects of iron on kidney function in the common population. Serum iron may be in improving cardiac function, renal function, at the same time, improve the pulmonary function.

Many studies have shown that serum iron plays an essential role in the pathophysiology of many diseases, which is the reason serum iron was selected as the independent variable.^[27–30]^ FEV1, FVC, and FEF 25%–75% were selected as the outcome variables of the study because these 3 indicators are not only commonly used in clinical practice but also reflect the pulmonary function more accurately.^[31,32]^ Serum iron concentration was found to be positively correlated with pulmonary function, which may be related to age, gender, race, and other factors. After controlling for the effects of the relevant single factors in the multiple regression analysis, the results still revealed a linear positive association between serum iron and pulmonary function. The results of the smooth fitting curve also confirm the positive associations between serum iron concentration and pulmonary function.

It was found that the bone morphogenetic protein pathway regulates serum iron concentrations by regulating hepcidin protein, a key protein in the regulation of iron metabolism.^[23,33,34]^ When hepcidin is elevated, it leads to decreased iron uptake and extracellular transport and decreased serum iron concentration, resulting in increased levels of oxidative stress and impaired function of the vascular endothelium. Iron is a strong redox agent and has an important role in protecting vascular endothelial stability. Hypoxia and inflammatory factors induce upregulation of hepcidin. Iron deficiency may be involved in hypoxic pulmonary hypertensive vascular injury by upregulating pulmonary artery endothelial cell levels and promoting apoptosis of endothelial cells and proliferation of lung artery smooth muscle cells, and further upregulation of hepcidin levels leads to a vicious cycle of iron deficiency.^[35]^ It is believed that serum iron concentration affects pulmonary function. Existing research shows that the iron in the pulmonary vascular function perception of oxygen plays an essential role. Decker et al^[36]^ demonstrated that the abnormal level of serum iron deficiency is strongly associated with the clinical severity of pulmonary hypertension; Livesey et al^[37]^ showed that insufficient or lost serum iron is associated with deep vein thrombosis and pulmonary embolism. Viethen et al^[38]^ treated patients with iron deficiency symptoms in pulmonary hypertension with intravenous iron therapy, resulting in a marked improvement in quality of life and the 6-minute walk test compared with the untreated group. Iron deficiency in anemic COPD patients is correlated with a moderate increase in systolic pulmonary artery pressure and a limitation of diffusion capability; in Eisenmenger patients, iron deficiency affects heart function as well as pulmonary function.^[29]^ Iron supplementation has been shown to improve symptoms and quality of life in patients with pulmonary hypertension and heart failure.^[8]^ Vaugier et al^[9]^ found that elevated serum iron levels in patients may improve the prognosis of renal transplantation and improve renal function and that the improvement in renal function, which alters the intrinsic mechanisms of circulating blood flow, also affects liver function and pulmonary function. None of these studies indicate whether serum iron concentration directly affects pulmonary function. A study by Sato et al^[39]^ demonstrated that elevated serum iron levels can counteract the harmful effects of smoking on pulmonary function in Japanese men. This suggests that serum iron could have a significant impact on lung function. However, only male smokers participated in the study and there was no comparison with female or nonsmokers. A study by Zhang et al investigated whether iron supplementation could exacerbate smoking-related pulmonary iron accumulation and increase the risk of infection and inflammation in these patients. Zhang et al concluded that further research should investigate how iron affects lung function. The authors also suggested that iron may be a new goal in COPD treatment.^[40]^ McKeever et al^[10]^ demonstrated that trace iron was independently associated with higher FEV1, while Ghio et al^[41]^ found that decreases in serum iron levels were independently associated with decreases in FVC and FEV1. The results of this study provide evidence that serum iron levels may be protective factors for pulmonary function and, therefore, serum iron levels may be a simple and accurate blood test to assess pulmonary function.

The present study included 5319 participants. This study has the advantage of large sample size, wide study population, and rigorous statistical methods. The results of confirmed that serum iron concentration was positively correlated with pulmonary function and that serum iron is a favorable factor for pulmonary function. Regulation of pulmonary function by serum iron levels may be a possibility in the future and provide guidelines for predicting pulmonary function in specific populations. Currently, however, this study can be used as a guide to improve pulmonary function in pre- and postoperative patients. Serum iron not only affects renal function and cardiovascular system but also significantly affects pulmonary function. To our knowledge, this is the first study to demonstrate a positive correlation between serum iron levels and pulmonary function. Simultaneously, the study also demonstrated that the combination of alcohol and dietary factors can enhance lung function to some extent. Furthermore, serum iron concentration is advantageous for improving lung function in diabetic patients.

This study has some limitations, first, the sample itself has limitations; the participants limited to the American population, and results may be different for other regions. Second, although the study controlled for relevant complex factor variables, other factors cannot be excluded. Third, the molecular mechanisms underlying the effects of serum iron on pulmonary function are unclear and require further basic research. Fourth, this study did not consider whether serum iron in patients with impaired pulmonary function. Finally, the present study did not consider whether serum iron affects patients with impaired pulmonary function.

## 5. Conclusions

Serum iron levels are protective factors for pulmonary function. Serum iron levels may provide a simple and accurate blood test to assess pulmonary function, providing a guide to predict pulmonary function in specific populations. Appropriate serum iron supplementation may improve pulmonary function.

## Acknowledgments

We sincerely thank all the data collection and gathering staff of NHANES database.

## Author contributions

**Data curation:** Lang Su, Silin Wang.

**Writing – original draft:** Lang Su, Yiping Wei.

**Writing – review & editing:** Lang Su, Yiping Wei.

**Resources:** Sheng Hu.

**Software:** Sheng Hu.

**Conceptualization:** Silin Wang.

**Methodology:** Qiang Guo, Yiping Wei.

**Project administration:** Qiang Guo, Yiping Wei.

**Funding acquisition:** Yiping Wei.

**Supervision:** Yiping Wei.

## Supplementary Material








