# Supplementary material for: Correlation between serum iron levels and pulmonary function: A cross-sectional analysis based on NHANES database 5319 cases
Source: Medicine (Baltimore). 2023 Dec 15;102(50):e36449. doi: 10.1097/MD.0000000000036449 (PMC10727669; doi:10.1097/MD.0000000000036449)
Supplement: Supplementary file 1 [file medi-102-e36449-s001.docx]

**Table S1. Multiple regression equation analysis for all subgroup variables**

| **Exposure** | **Non-adjusted** | **Adjust I** | **Adjust II** |
| --- | --- | --- | --- |
| ***Stratification by age*** |  |  |  |
| Age (years)= < 40 |  |  |  |
| Baseline FVC (mL) |  |  |  |
| Iron,refigerated (umol/L) | 28.15 (22.58, 33.72) <0.0001 | 5.23 (0.77, 9.69) 0.0216 | 5.99 (2.42, 9.56) 0.0010 |
| Iron,refigerated (umol/L) Tertile |  |  |  |
| Low | 0 | 0 | 0 |
| Medium | 275.72 (184.41, 367.03) <0.0001 | 8.07 (-63.78, 79.93) 0.8257 | 14.84 (-41.91, 71.58) 0.6083 |
| High | 450.02 (361.31, 538.74) <0.0001 | 82.47 (11.38, 153.55) 0.0230 | 102.67 (45.92, 159.41) 0.0004 |
| Baseline FEV 1 (mL) |  |  |  |
| Iron,refigerated (umol/L) | 23.31 (18.85, 27.76) <0.0001 | 6.02 (2.30, 9.74) 0.0015 | 6.12 (2.96, 9.28) 0.0001 |
| Iron,refigerated (umol/L) Tertile |  |  |  |
| Low | 0 | 0 | 0 |
| Medium | 207.43 (134.35, 280.50) <0.0001 | 5.15 (-54.85, 65.16) 0.8663 | 10.03 (-40.19, 60.25) 0.6954 |
| High | 363.26 (292.27, 434.25) <0.0001 | 84.15 (24.79, 143.51) 0.0055 | 94.55 (44.33, 144.77) 0.0002 |
| Baseline FEF 25-75% (mL/s) |  |  |  |
| Iron,refigerated (umol/L) | 19.43 (12.96, 25.90) <0.0001 | 6.34 (-0.02, 12.70) 0.0509 | 6.25 (0.06, 12.44) 0.0480 |
| Iron,refigerated (umol/L) Tertile |  |  |  |
| Low | 0 | 0 | 0 |
| Medium | 154.47 (48.26, 260.68) 0.0044 | 0.14 (-102.36, 102.64) 0.9979 | 9.19 (-89.28, 107.65) 0.8549 |
| High | 290.46 (187.27, 393.64) <0.0001 | 74.63 (-26.77, 176.03) 0.1493 | 84.22 (-14.25, 182.69) 0.0938 |
| Age (years)= >= 40 , <60 |  |  |  |
| Baseline FVC (mL) |  |  |  |
| Iron,refigerated (umol/L) | 36.73 (29.23, 44.22) <0.0001 | 7.78 (2.38, 13.18) 0.0048 | 4.47 (-0.37, 9.31) 0.0705 |
| Iron,refigerated (umol/L) Tertile |  |  |  |
| Low | 0 | 0 | 0 |
| Medium | 323.63 (205.45, 441.80) <0.0001 | 55.75 (-27.07, 138.56) 0.1872 | 36.33 (-37.39, 110.04) 0.3342 |
| High | 547.77 (429.19, 666.34) <0.0001 | 109.08 (24.00, 194.16) 0.0121 | 48.90 (-27.17, 124.97) 0.2079 |
| Baseline FEV 1 (mL) |  |  |  |
| Iron,refigerated (umol/L) | 28.50 (22.78, 34.23) <0.0001 | 8.05 (3.63, 12.47) 0.0004 | 6.32 (2.24, 10.40) 0.0024 |
| Iron,refigerated (umol/L) Tertile |  |  |  |
| Low | 0 | 0 | 0 |
| Medium | 236.96 (146.86, 327.07) <0.0001 | 45.92 (-21.81, 113.65) 0.1841 | 32.88 (-29.20, 94.96) 0.2994 |
| High | 434.76 (344.35, 525.16) <0.0001 | 124.72 (55.13, 194.30) 0.0005 | 90.26 (26.20, 154.33) 0.0058 |
| Baseline FEF 25-75% (mL/s) |  |  |  |
| Iron,refigerated (umol/L) | 22.27 (14.11, 30.42) <0.0001 | 9.90 (1.91, 17.89) 0.0152 | 10.92 (2.97, 18.86) 0.0071 |
| Iron,refigerated (umol/L) Tertile |  |  |  |
| Low | 0 | 0 | 0 |
| Medium | 154.30 (26.18, 282.42) 0.0184 | 32.37 (-89.93, 154.67) 0.6040 | 29.39 (-91.44, 150.21) 0.6336 |
| High | 359.87 (231.33, 488.42) <0.0001 | 172.66 (47.01, 298.30) 0.0072 | 180.45 (55.77, 305.13) 0.0046 |
| Age (years)= >= 60 |  |  |  |
| Baseline FVC (mL) |  |  |  |
| Iron,refigerated (umol/L) | 42.25 (32.50, 52.01) <0.0001 | 14.78 (7.47, 22.08) <0.0001 | 8.88 (2.15, 15.62) 0.0099 |
| Iron,refigerated (umol/L) Tertile |  |  |  |
| Low | 0 | 0 | 0 |
| Medium | 302.99 (170.89, 435.09) <0.0001 | 121.20 (24.94, 217.46) 0.0137 | 72.12 (-15.90, 160.14) 0.1086 |
| High | 599.00 (457.16, 740.84) <0.0001 | 214.64(108.48,320.81)<0.0001 | 118.17 (20.06, 216.28) 0.0184 |
| Baseline FEV 1 (mL) |  |  |  |
| Iron,refigerated (umol/L) | 26.34 (19.10, 33.58) <0.0001 | 8.72 (2.67, 14.77) 0.0048 | 4.49 (-1.23, 10.22) 0.1245 |
| Iron,refigerated (umol/L) Tertile |  |  |  |
| Low | 0 | 0 | 0 |
| Medium | 181.82 (83.87, 279.78) 0.0003 | 67.31 (-12.37, 146.99) 0.0981 | 39.76 (-35.05, 114.57) 0.2978 |
| High | 378.10 (272.92, 483.28) <0.0001 | 133.91 (46.03, 221.79) 0.0029 | 67.75 (-15.64, 151.14) 0.1116 |
| Baseline FEF 25-75% (mL/s) |  |  |  |
| Iron,refigerated (umol/L) | 6.26 (-3.50, 16.02) 0.2090 | 0.04 (-9.90, 9.98) 0.9939 | -2.65 (-12.67, 7.37) 0.6044 |
| Iron,refigerated (umol/L) Tertile |  |  |  |
| Low | 0 | 0 | 0 |
| Medium | 10.57 (-121.41, 142.54) 0.8753 | -27.20(-158.14, 103.74) 0.6840 | -27.24 (-158.13, 103.65) 0.6834 |
| High | 84.81 (-56.90, 226.52) 0.2411 | 1.10 (-143.32, 145.52) 0.9881 | -27.99 (-173.90, 117.91) 0.7070 |
| Total |  |  |  |
| Baseline FVC (mL) |  |  |  |
| Iron,refigerated (umol/L) | 33.01 (28.94, 37.07) <0.0001 | 7.52 (4.40, 10.64) <0.0001 | 5.45 (2.78, 8.13) <0.0001 |
| Iron,refigerated (umol/L) Tertile |  |  |  |
| Low | 0 | 0 | 0 |
| Medium | 295.00 (231.55, 358.45) <0.0001 | 45.43 (-2.30, 93.16) 0.0622 | 20.50 (-19.94, 60.94) 0.3204 |
| High | 506.13 (442.65, 569.61) <0.0001 | 115.07(66.31, 163.82) <0.0001 | 79.32 (37.65, 120.99) 0.0002 |
| Baseline FEV 1 (mL) |  |  |  |
| Iron,refigerated (umol/L) | 25.35 (22.19, 28.51) <0.0001 | 7.07 (4.48, 9.66) <0.0001 | 5.80 (3.49, 8.11) <0.0001 |
| Iron,refigerated (umol/L) Tertile |  |  |  |
| Low | 0 | 0 | 0 |
| Medium | 210.37 (161.00, 259.74) <0.0001 | 30.63 (-8.97, 70.23) 0.1295 | 16.22 (-18.77, 51.21) 0.3636 |
| High | 386.51 (337.12, 435.90) <0.0001 | 104.55(64.10, 145.00) <0.0001 | 82.40 (46.34, 118.45) <0.0001 |
| Baseline FEF 25-75% (mL/s) |  |  |  |
| Iron,refigerated (umol/L) | 18.13 (13.62, 22.64) <0.0001 | 6.39 (1.92, 10.85) 0.0051 | 6.72 (2.30, 11.13) 0.0029 |
| Iron,refigerated (umol/L) Tertile |  |  |  |
| Low | 0 | 0 | 0 |
| Medium | 123.78 (53.31, 194.25) 0.0006 | 5.63 (-62.66, 73.93) 0.8715 | 8.10 (-58.71, 74.92) 0.8121 |
| High | 271.26 (200.76, 341.76) <0.0001 | 86.07 (16.31, 155.84) 0.0156 | 89.96 (21.12, 158.81) 0.0105 |
| ***Stratification by gender*** |  |  |  |
| Gender= Male |  |  |  |
| Baseline FVC (mL) |  |  |  |
| Iron,refigerated (umol/L) | 19.47 (13.69, 25.26) <0.0001 | 12.49 (7.35, 17.62) <0.0001 | 9.44 (5.17, 13.72) <0.0001 |
| Iron,refigerated (umol/L) Tertile |  |  |  |
| Low | 0 | 0 | 0 |
| Medium | 140.02 (43.02, 237.02) 0.0047 | 101.43 (16.10, 186.76) 0.0199 | 65.37 (-5.38, 136.12) 0.0702 |
| High | 298.65 (204.62, 392.68) <0.0001 | 196.50(113.21,279.79)<0.0001 | 135.10 (65.63, 204.56) 0.0001 |
| Baseline FEV 1 (mL) |  |  |  |
| Iron,refigerated (umol/L) | 17.62 (12.53, 22.71) <0.0001 | 11.28 (7.04, 15.51) <0.0001 | 9.27 (5.58, 12.97) <0.0001 |
| Iron,refigerated (umol/L) Tertile |  |  |  |
| Low | 0 | 0 | 0 |
| Medium | 90.41 (5.05, 175.77) 0.0380 | 60.42 (-10.05, 130.88) 0.0930 | 36.79 (-24.38, 97.97) 0.2386 |
| High | 258.21 (175.46, 340.96) <0.0001 | 162.71(93.92, 231.50) <0.0001 | 121.04 (60.97, 181.11) <0.0001 |
| Baseline FEF 25-75% (mL/s) |  |  |  |
| Iron,refigerated (umol/L) | 19.25 (10.87, 27.63) <0.0001 | 12.20 (5.20, 19.20) 0.0006 | 11.51 (4.63, 18.38) 0.0010 |
| Iron,refigerated (umol/L) Tertile |  |  |  |
| Low | 0 | 0 | 0 |
| Medium | 31.40 (-109.08, 171.89) 0.6613 | 10.37 (-106.01, 126.76) 0.8613 | -2.72 (-116.41, 110.97) 0.9626 |
| High | 250.68 (114.49, 386.88) 0.0003 | 140.59 (26.99, 254.19) 0.0153 | 118.74 (7.09, 230.38) 0.0372 |
| Gender= Female |  |  |  |
| Baseline FVC (mL) |  |  |  |
| Iron,refigerated (umol/L) | 9.34 (4.96, 13.71) <0.0001 | 2.70 (-0.75, 6.15) 0.1251 | 1.11 (-1.94, 4.17) 0.4746 |
| Iron,refigerated (umol/L) Tertile |  |  |  |
| Low | 0 | 0 | 0 |
| Medium | 27.27 (-35.69, 90.24) 0.3959 | 19.39 (-30.00, 68.78) 0.4416 | -1.00 (-43.96, 41.96) 0.9637 |
| High | 163.53 (96.33, 230.72) <0.0001 | 45.00 (-8.14, 98.13) 0.0971 | 24.26 (-22.65, 71.17) 0.3108 |
| Baseline FEV 1 (mL) |  |  |  |
| Iron,refigerated (umol/L) | 7.98 (4.01, 11.95) <0.0001 | 2.84 (-0.07, 5.75) 0.0556 | 1.78 (-0.88, 4.43) 0.1893 |
| Iron,refigerated (umol/L) Tertile |  |  |  |
| Low | 0 | 0 | 0 |
| Medium | 10.34 (-46.75, 67.44) 0.7226 | 18.98 (-22.60, 60.56) 0.3711 | 7.29 (-30.06, 44.64) 0.7020 |
| High | 149.07 (88.14, 210.01) <0.0001 | 50.55 (5.82, 95.29) 0.0269 | 37.80 (-2.99, 78.58) 0.0694 |
| Baseline FEF 25-75% (mL/s) |  |  |  |
| Iron,refigerated (umol/L) | 3.98 (-2.77, 10.73) 0.2484 | 0.29 (-5.17, 5.75) 0.9179 | 0.90 (-4.55, 6.35) 0.7458 |
| Iron,refigerated (umol/L) Tertile |  |  |  |
| Low | 0 | 0 | 0 |
| Medium | -36.58 (-133.67, 60.52) 0.4603 | 10.56 (-67.56, 88.67) 0.7911 | 18.93 (-57.77, 95.63) 0.6287 |
| High | 117.85 (14.23, 221.48) 0.0259 | 27.74 (-56.30, 111.79) 0.5177 | 42.20 (-41.55, 125.95) 0.3235 |
| Total |  |  |  |
| Baseline FVC (mL) |  |  |  |
| Iron,refigerated (umol/L) | 14.53 (10.88, 18.17) <0.0001 | 7.52 (4.40, 10.64) <0.0001 | 5.45 (2.78, 8.13) <0.0001 |
| Iron,refigerated (umol/L) Tertile |  |  |  |
| Low | 0 | 0 | 0 |
| Medium | 72.22 (16.14, 128.31) 0.0116 | 45.43 (-2.30, 93.16) 0.0622 | 20.50 (-19.94, 60.94) 0.3204 |
| High | 224.97 (168.13, 281.81) <0.0001 | 115.07(66.31, 163.82) <0.0001 | 79.32 (37.65, 120.99) 0.0002 |
| Baseline FEV 1 (mL) |  |  |  |
| Iron,refigerated (umol/L) | 12.92 (9.68, 16.16) <0.0001 | 7.07 (4.48, 9.66) <0.0001 | 5.80 (3.49, 8.11) <0.0001 |
| Iron,refigerated (umol/L) Tertile |  |  |  |
| Low | 0 | 0 | 0 |
| Medium | 41.52 (-8.37, 91.40) 0.1029 | 30.63 (-8.97, 70.23) 0.1295 | 16.22 (-18.77, 51.21) 0.3636 |
| High | 199.72 (149.17, 250.27) <0.0001 | 104.55(64.10, 145.00) <0.0001 | 82.40 (46.34, 118.45) <0.0001 |
| Baseline FEF 25-75% (mL/s) |  |  |  |
| Iron,refigerated (umol/L) | 11.80 (6.40, 17.20) <0.0001 | 6.39 (1.92, 10.85) 0.0051 | 6.72 (2.30, 11.13) 0.0029 |
| Iron,refigerated (umol/L) Tertile |  |  |  |
| Low | 0 | 0 | 0 |
| Medium | -12.39 (-95.47, 70.69) 0.7700 | 5.63 (-62.66, 73.93) 0.8715 | 8.10 (-58.71, 74.92) 0.8121 |
| High | 182.22 (98.02, 266.42) <0.0001 | 86.07 (16.31, 155.84) 0.0156 | 89.96 (21.12, 158.81) 0.0105 |
| ***Stratification by race*** | |  |  |
| Race/Hispanic origin= Mexican American | |  |  |
| Baseline FVC (mL) |  |  |  |
| Iron,refigerated (umol/L) | 29.55 (20.73, 38.38) <0.0001 | 7.65 (1.03, 14.27) 0.0237 | 4.31 (-1.18, 9.81) 0.1241 |
| Iron,refigerated (umol/L) Tertile |  |  |  |
| Low | 0 | 0 | 0 |
| Medium | 152.36 (11.40, 293.32) 0.0343 | -12.24 (-115.62, 91.14) 0.8165 | -35.33 (-120.38, 49.72) 0.4157 |
| High | 467.58 (329.13, 606.02) <0.0001 | 132.00 (28.46, 235.53) 0.0126 | 50.12 (-36.43, 136.67) 0.2566 |
| Baseline FEV 1 (mL) |  |  |  |
| Iron,refigerated (umol/L) | 21.70 (14.31, 29.10) <0.0001 | 5.92 (0.54, 11.30) 0.0312 | 3.69 (-0.92, 8.30) 0.1167 |
| Iron,refigerated (umol/L) Tertile |  |  |  |
| Low | 0 | 0 | 0 |
| Medium | 102.32 (-15.67, 220.32) 0.0895 | -4.93 (-88.95, 79.08) 0.9084 | -21.92 (-93.21, 49.38) 0.5469 |
| High | 350.00 (234.11, 465.89) <0.0001 | 107.69 (23.55, 191.83) 0.0123 | 50.18 (-22.37, 122.74) 0.1755 |
| Baseline FEF 25-75% (mL/s) |  |  |  |
| Iron,refigerated (umol/L) | 12.54 (1.57, 23.51) 0.0253 | 3.01 (-6.17, 12.18) 0.5208 | 2.78 (-6.24, 11.80) 0.5460 |
| Iron,refigerated (umol/L) Tertile |  |  |  |
| Low | 0 | 0 | 0 |
| Medium | 8.36 (-166.67, 183.40) 0.9254 | -26.13(-169.53, 117.26) 0.7210 | -29.53 (-169.11, 110.06) 0.6785 |
| High | 234.59 (62.67, 406.50) 0.0076 | 86.89 (-56.72, 230.50) 0.2359 | 65.30 (-76.74, 207.35) 0.3677 |
| Race/Hispanic origin= Other Hispanic |  |  |  |
| Baseline FVC (mL) |  |  |  |
| Iron,refigerated (umol/L) | 37.28 (25.08, 49.48) <0.0001 | 9.55 (0.74, 18.36) 0.0340 | 7.15 (-0.46, 14.77) 0.0661 |
| Iron,refigerated (umol/L) Tertile |  |  |  |
| Low | 0 | 0 | 0 |
| Medium | 408.44 (219.86, 597.02) <0.0001 | 86.77 (-48.84, 222.38) 0.2103 | 18.73 (-96.58, 134.04) 0.7503 |
| High | 635.33 (443.52, 827.15) <0.0001 | 161.30 (21.14, 301.45) 0.0245 | 105.20 (-14.76, 225.16) 0.0862 |
| Baseline FEV 1 (mL) |  |  |  |
| Iron,refigerated (umol/L) | 29.92 (19.81, 40.03) <0.0001 | 6.99 (-0.01, 13.99) 0.0509 | 5.55 (-0.63, 11.73) 0.0787 |
| Iron,refigerated (umol/L) Tertile |  |  |  |
| Low | 0 | 0 | 0 |
| Medium | 307.58 (151.19, 463.96) 0.0001 | 64.97 (-42.80, 172.75) 0.2378 | 16.85 (-76.69, 110.39) 0.7242 |
| High | 511.45 (352.39, 670.51) <0.0001 | 117.78 (6.39, 229.17) 0.0387 | 85.41 (-11.90, 182.72) 0.0859 |
| Baseline FEF 25-75% (mL/s) |  |  |  |
| Iron,refigerated (umol/L) | 24.24 (9.95, 38.53) 0.0009 | 2.53 (-9.23, 14.29) 0.6734 | 3.14 (-8.67, 14.95) 0.6025 |
| Iron,refigerated (umol/L) Tertile |  |  |  |
| Low | 0 | 0 | 0 |
| Medium | 191.81 (-30.08, 413.69) 0.0907 | 12.17 (-169.00, 193.33) 0.8953 | -11.39 (-190.20, 167.42) 0.9007 |
| High | 414.88 (189.19, 640.57) 0.0003 | 37.88 (-149.36, 225.12) 0.6918 | 41.34 (-144.68, 227.35) 0.6633 |
| Race/Hispanic origin= Non-Hispanic white | |  |  |
| Baseline FVC (mL) |  |  |  |
| Iron,refigerated (umol/L) | 31.33 (24.57, 38.09) <0.0001 | 7.63 (2.86, 12.41) 0.0017 | 5.15 (1.06, 9.24) 0.0136 |
| Iron,refigerated (umol/L) Tertile |  |  |  |
| Low | 0 | 0 | 0 |
| Medium | 282.43 (174.35, 390.51) <0.0001 | 88.73 (13.50, 163.95) 0.0209 | 46.90 (-16.93, 110.72) 0.1500 |
| High | 489.97 (383.37, 596.58) <0.0001 | 118.54 (43.35, 193.74) 0.0020 | 76.50 (12.20, 140.81) 0.0198 |
| Baseline FEV 1 (mL) |  |  |  |
| Iron,refigerated (umol/L) | 26.14 (20.44, 31.85) <0.0001 | 8.04 (4.07, 12.00) <0.0001 | 6.57 (3.01, 10.14) 0.0003 |
| Iron,refigerated (umol/L) Tertile |  |  |  |
| Low | 0 | 0 | 0 |
| Medium | 190.73 (99.46, 282.00) <0.0001 | 54.35 (-8.16, 116.87) 0.0885 | 34.45 (-21.23, 90.14) 0.2254 |
| High | 396.05 (306.03, 486.08) <0.0001 | 110.16 (47.67, 172.65) 0.0006 | 86.26 (30.15, 142.37) 0.0026 |
| Baseline FEF 25-75% (mL/s) |  |  |  |
| Iron,refigerated (umol/L) | 24.70 (16.36, 33.04) <0.0001 | 9.16 (2.50, 15.83) 0.0071 | 9.59 (2.97, 16.21) 0.0045 |
| Iron,refigerated (umol/L) Tertile |  |  |  |
| Low | 0 | 0 | 0 |
| Medium | 132.04 (-1.38, 265.46) 0.0525 | 41.41 (-63.76, 146.57) 0.4404 | 50.80 (-52.54, 154.14) 0.3354 |
| High | 353.28 (221.68, 484.88) <0.0001 | 103.88 (-1.23, 209.00) 0.0529 | 111.55 (7.43, 215.68) 0.0359 |
| Race/Hispanic origin= Non-Hispanic black | |  |  |
| Baseline FVC (mL) |  |  |  |
| Iron,refigerated (umol/L) | 28.46 (18.27, 38.65) <0.0001 | 4.01 (-3.69, 11.71) 0.3075 | 1.35 (-5.45, 8.15) 0.6975 |
| Iron,refigerated (umol/L) Tertile |  |  |  |
| Low | 0 | 0 | 0 |
| Medium | 189.00 (53.56, 324.45) 0.0064 | 12.34 (-88.47, 113.15) 0.8104 | 12.27 (-76.73, 101.28) 0.7870 |
| High | 424.07 (271.48, 576.65) <0.0001 | 51.70 (-63.52, 166.93) 0.3793 | 35.18 (-66.64, 137.00) 0.4984 |
| Baseline FEV 1 (mL) |  |  |  |
| Iron,refigerated (umol/L) | 23.11 (14.20, 32.01) <0.0001 | 4.75 (-1.93, 11.43) 0.1635 | 2.85 (-3.32, 9.02) 0.3656 |
| Iron,refigerated (umol/L) Tertile |  |  |  |
| Low | 0 | 0 | 0 |
| Medium | 124.26 (6.12, 242.40) 0.0395 | 11.67 (-75.75, 99.10) 0.7936 | 9.66 (-71.05, 90.36) 0.8147 |
| High | 364.58 (231.49, 497.67) <0.0001 | 76.54 (-23.39, 176.46) 0.1336 | 66.69 (-25.63, 159.02) 0.1572 |
| Baseline FEF 25-75% (mL/s) |  |  |  |
| Iron,refigerated (umol/L) | 20.73 (6.01, 35.46) 0.0059 | 6.13 (-6.79, 19.04) 0.3527 | 5.30 (-7.56, 18.16) 0.4195 |
| Iron,refigerated (umol/L) Tertile |  |  |  |
| Low | 0 | 0 | 0 |
| Medium | 12.02 (-183.41, 207.45) 0.9041 | -33.28(-202.28, 135.72) 0.6996 | -37.04 (-205.20, 131.11) 0.6660 |
| High | 337.31 (117.15, 557.46) 0.0027 | 87.86 (-105.30, 281.02) 0.3729 | 100.42 (-91.95, 292.79) 0.3065 |
| Race/Hispanic origin= Other races - Including multi-racial | |  |  |
| Baseline FVC (mL) |  |  |  |
| Iron,refigerated (umol/L) | 33.26 (17.70, 48.82) <0.0001 | 14.13 (1.74, 26.53) 0.0262 | 17.65 (7.03, 28.28) 0.0013 |
| Iron,refigerated (umol/L) Tertile |  |  |  |
| Low | 0 | 0 | 0 |
| Medium | 130.61 (-158.40, 419.62) 0.3765 | 29.04 (-193.83, 251.91) 0.7986 | 126.24 (-67.68, 320.16) 0.2031 |
| High | 397.21 (128.64, 665.79) 0.0040 | 174.94 (-34.16, 384.05) 0.1022 | 255.64 (71.27, 440.01) 0.0070 |
| Baseline FEV 1 (mL) |  |  |  |
| Iron,refigerated (umol/L) | 27.11 (13.03, 41.19) 0.0002 | 12.08 (1.65, 22.51) 0.0240 | 14.83 (5.40, 24.27) 0.0023 |
| Iron,refigerated (umol/L) Tertile |  |  |  |
| Low | 0 | 0 | 0 |
| Medium | 74.69 (-186.15, 335.53) 0.5751 | 20.95 (-166.52, 208.42) 0.8268 | 90.83 (-81.01, 262.67) 0.3011 |
| High | 315.02 (72.62, 557.42) 0.0114 | 154.96 (-20.93, 330.85) 0.0853 | 220.03 (56.65, 383.41) 0.0088 |
| Baseline FEF 25-75% (mL/s) |  |  |  |
| Iron,refigerated (umol/L) | 22.32 (1.27, 43.37) 0.0386 | 9.17 (-7.30, 25.65) 0.2762 | 13.75 (-2.94, 30.45) 0.1075 |
| Iron,refigerated (umol/L) Tertile |  |  |  |
| Low | 0 | 0 | 0 |
| Medium | 0.21 (-386.43, 386.85) 0.9991 | 7.65 (-287.65, 302.95) 0.9595 | 86.94 (-215.54, 389.42) 0.5737 |
| High | 255.14 (-104.16, 614.44) 0.1651 | 146.91(-130.15,423.97) 0.2996 | 257.13 (-30.46, 544.72) 0.0809 |
| Total |  |  |  |
| Baseline FVC (mL) |  |  |  |
| Iron,refigerated (umol/L) | 31.33 (27.05, 35.60) <0.0001 | 7.52 (4.40, 10.64) <0.0001 | 5.45 (2.78, 8.13) <0.0001 |
| Iron,refigerated (umol/L) Tertile |  |  |  |
| Low | 0 | 0 | 0 |
| Medium | 243.07 (176.75, 309.39) <0.0001 | 45.43 (-2.30, 93.16) 0.0622 | 20.50 (-19.94, 60.94) 0.3204 |
| High | 484.58 (417.82, 551.35) <0.0001 | 115.07(66.31, 163.82) <0.0001 | 79.32 (37.65, 120.99) 0.0002 |
| Baseline FEV 1 (mL) |  |  |  |
| Iron,refigerated (umol/L) | 25.21 (21.58, 28.83) <0.0001 | 7.07 (4.48, 9.66) <0.0001 | 5.80 (3.49, 8.11) <0.0001 |
| Iron,refigerated (umol/L) Tertile |  |  |  |
| Low | 0 | 0 | 0 |
| Medium | 166.71 (110.44, 222.98) <0.0001 | 30.63 (-8.97, 70.23) 0.1295 | 16.22 (-18.77, 51.21) 0.3636 |
| High | 388.30 (331.65, 444.94) <0.0001 | 104.55(64.10, 145.00) <0.0001 | 82.40 (46.34, 118.45) <0.0001 |
| Baseline FEF 25-75% (mL/s) |  |  |  |
| Iron,refigerated (umol/L) | 21.19 (15.77, 26.61) <0.0001 | 6.39 (1.92, 10.85) 0.0051 | 6.72 (2.30, 11.13) 0.0029 |
| Iron,refigerated (umol/L) Tertile |  |  |  |
| Low | 0 | 0 | 0 |
| Medium | 82.95 (-1.20, 167.11) 0.0534 | 5.63 (-62.66, 73.93) 0.8715 | 8.10 (-58.71, 74.92) 0.8121 |
| High | 322.50 (237.78, 407.21) <0.0001 | 86.07 (16.31, 155.84) 0.0156 | 89.96 (21.12, 158.81) 0.0105 |
| ***Stratification by education level*** |  |  |  |
| Education level= Less than 9th grade |  |  |  |
| Baseline FVC (mL) |  |  |  |
| Iron,refigerated (umol/L) | 30.40 (16.80, 44.00) <0.0001 | -2.93 (-12.27, 6.41) 0.5393 | -0.88 (-9.46, 7.70) 0.8406 |
| Iron,refigerated (umol/L) Tertile |  |  |  |
| Low | 0 | 0 | 0 |
| Medium | 279.99 (69.81, 490.16) 0.0093 | 99.55 (-39.34, 238.43) 0.1608 | 29.23 (-97.50, 155.96) 0.6515 |
| High | 500.31 (283.52, 717.11) <0.0001 | 30.29 (-117.64, 178.22) 0.6884 | 18.25 (-118.22, 154.73) 0.7933 |
| Baseline FEV 1 (mL) |  |  |  |
| Iron,refigerated (umol/L) | 25.64 (14.35, 36.93) <0.0001 | 0.34 (-7.47, 8.15) 0.9322 | 2.59 (-4.86, 10.05) 0.4960 |
| Iron,refigerated (umol/L) Tertile |  |  |  |
| Low | 0 | 0 | 0 |
| Medium | 224.95 (50.63, 399.26) 0.0118 | 90.74 (-25.28, 206.77) 0.1260 | 46.60 (-63.39, 156.60) 0.4067 |
| High | 427.57 (247.77, 607.38) <0.0001 | 74.41 (-49.17, 197.99) 0.2385 | 82.15 (-36.30, 200.60) 0.1747 |
| Baseline FEF 25-75% (mL/s) |  |  |  |
| Iron,refigerated (umol/L) | 31.32 (14.43, 48.20) 0.0003 | 11.12 (-3.45, 25.68) 0.1355 | 14.34 (-0.56, 29.25) 0.0599 |
| Iron,refigerated (umol/L) Tertile |  |  |  |
| Low | 0 | 0 | 0 |
| Medium | 206.44 (-54.65, 467.52) 0.1219 | 108.99(-107.61,325.59) 0.3245 | 109.65 (-110.12, 329.42) 0.3287 |
| High | 509.17 (239.87, 778.48) 0.0002 | 232.48 (1.77, 463.18) 0.0489 | 283.15 (46.47, 519.82) 0.0195 |
| Education level= 9-11th grade |  |  |  |
| Baseline FVC (mL) |  |  |  |
| Iron,refigerated (umol/L) | 38.85 (26.04, 51.66) <0.0001 | 9.47 (1.18, 17.76) 0.0255 | 6.02 (-1.49, 13.52) 0.1165 |
| Iron,refigerated (umol/L) Tertile |  |  |  |
| Low | 0 | 0 | 0 |
| Medium | 496.43 (300.92, 691.93) <0.0001 | 113.14 (-13.55, 239.83) 0.0806 | 70.17 (-44.32, 184.66) 0.2301 |
| High | 604.17 (400.50, 807.83) <0.0001 | 120.23 (-11.68, 252.14) 0.0745 | 69.08 (-50.15, 188.31) 0.2566 |
| Baseline FEV 1 (mL) |  |  |  |
| Iron,refigerated (umol/L) | 31.45 (20.80, 42.09) <0.0001 | 9.60 (2.72, 16.47) 0.0064 | 7.55 (1.14, 13.95) 0.0212 |
| Iron,refigerated (umol/L) Tertile |  |  |  |
| Low | 0 | 0 | 0 |
| Medium | 291.22 (128.16, 454.28) 0.0005 | 30.32 (-74.80, 135.44) 0.5721 | 3.03 (-94.67, 100.73) 0.9516 |
| High | 491.75 (321.88, 661.61) <0.0001 | 126.80 (17.35, 236.25) 0.0235 | 94.44 (-7.31, 196.18) 0.0694 |
| Baseline FEF 25-75% (mL/s) |  |  |  |
| Iron,refigerated (umol/L) | 26.33 (10.92, 41.74) 0.0009 | 8.81 (-3.64, 21.26) 0.1658 | 9.15 (-3.21, 21.51) 0.1473 |
| Iron,refigerated (umol/L) Tertile |  |  |  |
| Low | 0 | 0 | 0 |
| Medium | 71.04 (-164.64, 306.72) 0.5549 | -71.77(-261.50, 117.96) 0.4587 | -71.54 (-259.69, 116.61) 0.4564 |
| High | 419.36 (173.84, 664.87) 0.0009 | 121.04 (-76.51, 318.58) 0.2303 | 116.68 (-79.26, 312.61) 0.2436 |
| Education level= High school graduate |  |  |  |
| Baseline FVC (mL) |  |  |  |
| Iron,refigerated (umol/L) | 43.54 (32.71, 54.37) <0.0001 | 6.88 (-0.34, 14.10) 0.0620 | 3.98 (-2.64, 10.61) 0.2390 |
| Iron,refigerated (umol/L) Tertile |  |  |  |
| Low | 0 | 0 | 0 |
| Medium | 369.92 (204.83, 535.01) <0.0001 | 78.41 (-27.98, 184.81) 0.1489 | 33.93 (-63.86, 131.71) 0.4966 |
| High | 641.53 (474.40, 808.65) <0.0001 | 103.88 (-6.58, 214.34) 0.0656 | 42.49 (-59.48, 144.47) 0.4143 |
| Baseline FEV 1 (mL) |  |  |  |
| Iron,refigerated (umol/L) | 33.23 (24.11, 42.35) <0.0001 | 5.17 (-1.01, 11.35) 0.1012 | 3.04 (-2.84, 8.92) 0.3111 |
| Iron,refigerated (umol/L) Tertile |  |  |  |
| Low | 0 | 0 | 0 |
| Medium | 264.14 (125.49, 402.78) 0.0002 | 50.68 (-40.37, 141.73) 0.2755 | 15.49 (-71.26, 102.24) 0.7264 |
| High | 506.35 (366.00, 646.70) <0.0001 | 84.32 (-10.20, 178.85) 0.0807 | 40.53 (-49.94, 131.00) 0.3801 |
| Baseline FEF 25-75% (mL/s) |  |  |  |
| Iron,refigerated (umol/L) | 26.44 (13.31, 39.57) <0.0001 | 3.05 (-7.97, 14.06) 0.5880 | 1.34 (-9.77, 12.46) 0.8126 |
| Iron,refigerated (umol/L) Tertile |  |  |  |
| Low | 0 | 0 | 0 |
| Medium | 142.04 (-57.21, 341.30) 0.1627 | -14.33(-176.64, 147.97) 0.8626 | -44.37 (-208.29, 119.55) 0.5959 |
| High | 439.03 (237.32, 640.74) <0.0001 | 60.99 (-107.51, 229.50) 0.4782 | 30.41 (-140.53, 201.35) 0.7274 |
| Education level= Some college or AA degree | |  |  |
| Baseline FVC (mL) |  |  |  |
| Iron,refigerated (umol/L) | 39.81 (30.59, 49.04) <0.0001 | 8.07 (2.05, 14.09) 0.0087 | 5.37 (-0.25, 10.99) 0.0612 |
| Iron,refigerated (umol/L) Tertile |  |  |  |
| Low | 0 | 0 | 0 |
| Medium | 220.54 (79.02, 362.05) 0.0023 | 0.24 (-89.84, 90.32) 0.9959 | -9.51 (-92.42, 73.40) 0.8222 |
| High | 638.99 (498.20, 779.79) <0.0001 | 130.83 (38.30, 223.35) 0.0057 | 91.85 (5.70, 178.01) 0.0369 |
| Baseline FEV 1 (mL) |  |  |  |
| Iron,refigerated (umol/L) | 29.88 (22.30, 37.46) <0.0001 | 6.18 (1.22, 11.13) 0.0147 | 4.56 (-0.13, 9.26) 0.0570 |
| Iron,refigerated (umol/L) Tertile |  |  |  |
| Low | 0 | 0 | 0 |
| Medium | 169.71 (53.53, 285.89) 0.0043 | 15.00 (-59.14, 89.15) 0.6917 | 10.44 (-58.88, 79.75) 0.7679 |
| High | 488.79 (373.21, 604.38) <0.0001 | 109.85 (33.69, 186.00) 0.0048 | 84.87 (12.85, 156.90) 0.0211 |
| Baseline FEF 25-75% (mL/s) |  |  |  |
| Iron,refigerated (umol/L) | 19.32 (8.18, 30.46) 0.0007 | 1.36 (-7.81, 10.53) 0.7714 | 1.52 (-7.75, 10.79) 0.7476 |
| Iron,refigerated (umol/L) Tertile |  |  |  |
| Low | 0 | 0 | 0 |
| Medium | 121.38 (-49.76, 292.53) 0.1647 | 30.24 (-107.12, 167.60) 0.6662 | 40.86 (-96.11, 177.82) 0.5589 |
| High | 356.68 (186.41, 526.94) <0.0001 | 74.11 (-66.98, 215.19) 0.3034 | 76.03 (-66.29, 218.35) 0.2953 |
| Education level= College graduate or above | |  |  |
| Baseline FVC (mL) |  |  |  |
| Iron,refigerated (umol/L) | 30.35 (19.51, 41.20) <0.0001 | 7.97 (0.66, 15.27) 0.0328 | 6.49 (-0.25, 13.23) 0.0595 |
| Iron,refigerated (umol/L) Tertile |  |  |  |
| Low | 0 | 0 | 0 |
| Medium | 213.88 (43.99, 383.77) 0.0138 | 18.26 (-94.80, 131.32) 0.7517 | 17.54 (-86.30, 121.39) 0.7406 |
| High | 483.91 (315.64, 652.18) <0.0001 | 97.77 (-16.61, 212.15) 0.0942 | 79.43 (-25.68, 184.54) 0.1389 |
| Baseline FEV 1 (mL) |  |  |  |
| Iron,refigerated (umol/L) | 22.96 (14.15, 31.77) <0.0001 | 7.43 (1.35, 13.50) 0.0167 | 6.38 (0.64, 12.11) 0.0296 |
| Iron,refigerated (umol/L) Tertile |  |  |  |
| Low | 0 | 0 | 0 |
| Medium | 137.90 (-0.37, 276.18) 0.0509 | 8.11 (-86.00, 102.22) 0.8659 | 10.08 (-78.32, 98.47) 0.8232 |
| High | 349.58 (212.63, 486.54) <0.0001 | 78.05 (-17.15, 173.26) 0.1084 | 65.82 (-23.64, 155.29) 0.1496 |
| Baseline FEF 25-75% (mL/s) |  |  |  |
| Iron,refigerated (umol/L) | 14.31 (1.44, 27.17) 0.0295 | 6.64 (-4.02, 17.30) 0.2223 | 6.36 (-4.42, 17.14) 0.2477 |
| Iron,refigerated (umol/L) Tertile |  |  |  |
| Low | 0 | 0 | 0 |
| Medium | 31.89 (-170.12, 233.90) 0.7571 | -8.53 (-173.62, 156.56) 0.9193 | -3.69 (-169.74, 162.35) 0.9652 |
| High | 164.01 (-36.07, 364.09) 0.1085 | 20.30 (-146.72, 187.32) 0.8118 | 19.02 (-149.04, 187.08) 0.8245 |
| Education level= Unknown |  |  |  |
| Baseline FVC (mL) |  |  |  |
| Iron,refigerated (umol/L) | 24.83 (16.64, 33.02) <0.0001 | 7.34 (0.16, 14.53) 0.0455 | 8.60 (3.25, 13.94) 0.0017 |
| Iron,refigerated (umol/L) Tertile |  |  |  |
| Low | 0 | 0 | 0 |
| Medium | 185.44 (49.74, 321.15) 0.0075 | -15.58(-132.22, 101.07) 0.7936 | 44.96 (-39.93, 129.85) 0.2995 |
| High | 347.02 (216.19, 477.85) <0.0001 | 81.86 (-32.20, 195.92) 0.1598 | 129.26 (44.94, 213.59) 0.0027 |
| Baseline FEV 1 (mL) |  |  |  |
| Iron,refigerated (umol/L) | 22.25 (15.37, 29.13) <0.0001 | 8.74 (2.50, 14.99) 0.0062 | 8.31 (3.44, 13.17) 0.0008 |
| Iron,refigerated (umol/L) Tertile |  |  |  |
| Low | 0 | 0 | 0 |
| Medium | 166.67 (52.57, 280.76) 0.0043 | 10.08 (-91.39, 111.54) 0.8457 | 44.47 (-32.84, 121.78) 0.2598 |
| High | 306.58 (196.57, 416.58) <0.0001 | 98.84 (-0.38, 198.05) 0.0511 | 115.91 (39.11, 192.71) 0.0032 |
| Baseline FEF 25-75% (mL/s) |  |  |  |
| Iron,refigerated (umol/L) | 20.60 (10.65, 30.55) <0.0001 | 10.41 (0.51, 20.31) 0.0396 | 8.33 (-1.00, 17.66) 0.0803 |
| Iron,refigerated (umol/L) Tertile |  |  |  |
| Low | 0 | 0 | 0 |
| Medium | 159.21 (-5.37, 323.80) 0.0582 | 31.66 (-129.17, 192.49) 0.6997 | 45.46 (-102.74, 193.67) 0.5478 |
| High | 270.81 (112.13, 429.49) 0.0009 | 105.63 (-51.64, 262.89) 0.1883 | 94.89 (-52.33, 242.10) 0.2068 |
| Total |  |  |  |
| Baseline FVC (mL) |  |  |  |
| Iron,refigerated (umol/L) | 34.55 (30.25, 38.84) <0.0001 | 6.90 (3.86, 9.93) <0.0001 | 5.45 (2.78, 8.13) <0.0001 |
| Iron,refigerated (umol/L) Tertile |  |  |  |
| Low | 0 | 0 | 0 |
| Medium | 278.81 (211.64, 345.99) <0.0001 | 35.63 (-10.82, 82.09) 0.1328 | 20.50 (-19.94, 60.94) 0.3204 |
| High | 534.49 (467.36, 601.63) <0.0001 | 102.78(55.33, 150.22) <0.0001 | 79.32 (37.65, 120.99) 0.0002 |
| Baseline FEV 1 (mL) |  |  |  |
| Iron,refigerated (umol/L) | 27.39 (23.83, 30.95) <0.0001 | 6.69 (4.14, 9.25) <0.0001 | 5.80 (3.49, 8.11) <0.0001 |
| Iron,refigerated (umol/L) Tertile |  |  |  |
| Low | 0 | 0 | 0 |
| Medium | 200.76 (145.11, 256.40) <0.0001 | 26.66 (-12.41, 65.73) 0.1812 | 16.22 (-18.77, 51.21) 0.3636 |
| High | 423.97 (368.36, 479.59) <0.0001 | 97.36 (57.45, 137.27) <0.0001 | 82.40 (46.34, 118.45) <0.0001 |
| Baseline FEF 25-75% (mL/s) |  |  |  |
| Iron,refigerated (umol/L) | 21.88 (16.69, 27.07) <0.0001 | 6.21 (1.76, 10.66) 0.0063 | 6.72 (2.30, 11.13) 0.0029 |
| Iron,refigerated (umol/L) Tertile |  |  |  |
| Low | 0 | 0 | 0 |
| Medium | 118.79 (37.75, 199.83) 0.0041 | 7.48 (-60.56, 75.53) 0.8294 | 8.10 (-58.71, 74.92) 0.8121 |
| High | 339.45 (258.45, 420.45) <0.0001 | 83.37 (13.87, 152.87) 0.0188 | 89.96 (21.12, 158.81) 0.0105 |
| ***Stratification by surgery*** |  |  |  |
| Thoracic/abdominal surgery= Yes |  |  |  |
| Baseline FVC (mL) |  |  |  |
| Iron,refigerated (umol/L) | 42.83 (32.19, 53.47) <0.0001 | 17.32 (9.72, 24.93) <0.0001 | 10.93 (3.94, 17.92) 0.0022 |
| Iron,refigerated (umol/L) Tertile |  |  |  |
| Low | 0 | 0 | 0 |
| Medium | 211.40 (65.97, 356.83) 0.0045 | 79.53 (-22.14, 181.19) 0.1256 | 31.94 (-59.70, 123.58) 0.4946 |
| High | 659.38 (503.71, 815.05) <0.0001 | 278.47(167.04,389.89)<0.001 | 180.33 (77.85, 282.82) 0.0006 |
| Baseline FEV 1 (mL) |  |  |  |
| Iron,refigerated (umol/L) | 31.63 (22.98, 40.28) <0.0001 | 13.57 (7.33, 19.81) <0.0001 | 8.25 (2.41, 14.10) 0.0058 |
| Iron,refigerated (umol/L) Tertile |  |  |  |
| Low | 0 | 0 | 0 |
| Medium | 132.08 (13.86, 250.29) 0.0288 | 51.68 (-31.74, 135.10) 0.2250 | 16.37 (-60.30, 93.03) 0.6757 |
| High | 490.62 (364.09, 617.16) <0.0001 | 215.96(124.53,307.39)<0.0001 | 137.49 (51.76, 223.23) 0.0017 |
| Baseline FEF 25-75% (mL/s) |  |  |  |
| Iron,refigerated (umol/L) | 20.77 (8.35, 33.19) 0.0011 | 9.68 (-0.65, 20.01) 0.0666 | 5.26 (-5.17, 15.70) 0.3230 |
| Iron,refigerated (umol/L) Tertile |  |  |  |
| Low | 0 | 0 | 0 |
| Medium | 29.27 (-140.58, 199.13) 0.7356 | 14.88 (-123.44, 153.19) 0.8331 | -4.29 (-141.16, 132.58) 0.9510 |
| High | 352.95 (171.14, 534.77) 0.0002 | 169.00 (17.41, 320.60) 0.0291 | 112.18 (-40.89, 265.25) 0.1512 |
| Thoracic/abdominal surgery= No |  |  |  |
| Baseline FVC (mL) |  |  |  |
| Iron,refigerated (umol/L) | 31.81 (27.13, 36.49) <0.0001 | 5.64 (2.21, 9.07) 0.0013 | 4.57 (1.67, 7.47) 0.0020 |
| Iron,refigerated (umol/L) Tertile |  |  |  |
| Low | 0 | 0 | 0 |
| Medium | 288.14 (213.18, 363.09) <0.0001 | 37.43 (-16.36, 91.21) 0.1727 | 18.31 (-26.80, 63.43) 0.4263 |
| High | 495.01 (420.98, 569.04) <0.0001 | 81.60 (27.39, 135.80) 0.0032 | 62.04 (16.24, 107.85) 0.0080 |
| Baseline FEV 1 (mL) |  |  |  |
| Iron,refigerated (umol/L) | 25.01 (21.10, 28.92) <0.0001 | 5.71 (2.86, 8.56) <0.0001 | 5.32 (2.80, 7.85) <0.0001 |
| Iron,refigerated (umol/L) Tertile |  |  |  |
| Low | 0 | 0 | 0 |
| Medium | 198.56 (136.02, 261.11) <0.0001 | 24.97 (-19.73, 69.67) 0.2737 | 15.65 (-23.65, 54.95) 0.4352 |
| High | 390.45 (328.68, 452.23) <0.0001 | 80.38 (35.32, 125.43) 0.0005 | 72.36 (32.46, 112.26) 0.0004 |
| Baseline FEF 25-75% (mL/s) |  |  |  |
| Iron,refigerated (umol/L) | 19.06 (13.21, 24.90) <0.0001 | 5.35 (0.40, 10.30) 0.0342 | 6.60 (1.71, 11.49) 0.0082 |
| Iron,refigerated (umol/L) Tertile |  |  |  |
| Low | 0 | 0 | 0 |
| Medium | 97.56 (4.08, 191.03) 0.0408 | 1.15 (-76.45, 78.74) 0.9769 | 8.92 (-67.13, 84.97) 0.8182 |
| High | 297.03 (204.72, 389.35) <0.0001 | 64.69 (-13.51, 142.89) 0.1050 | 82.09 (4.88, 159.29) 0.0372 |
| Total |  |  |  |
| Baseline FVC (mL) |  |  |  |
| Iron,refigerated (umol/L) | 33.44 (29.16, 37.72) <0.0001 | 7.42 (4.30, 10.55) <0.0001 | 5.45 (2.78, 8.13) <0.0001 |
| Iron,refigerated (umol/L) Tertile |  |  |  |
| Low | 0 | 0 | 0 |
| Medium | 274.04 (207.26, 340.83) <0.0001 | 45.40 (-2.32, 93.12) 0.0623 | 20.50 (-19.94, 60.94) 0.3204 |
| High | 519.81 (452.94, 586.67) <0.0001 | 114.03 (65.28, 162.79) <0.0001 | 79.32 (37.65, 120.99) 0.0002 |
| Baseline FEV 1 (mL) |  |  |  |
| Iron,refigerated (umol/L) | 25.99 (22.43, 29.55) <0.0001 | 6.94 (4.35, 9.53) <0.0001 | 5.80 (3.49, 8.11) <0.0001 |
| Iron,refigerated (umol/L) Tertile |  |  |  |
| Low | 0 | 0 | 0 |
| Medium | 186.23 (130.74, 241.73) <0.0001 | 30.59 (-8.98, 70.16) 0.1297 | 16.22 (-18.77, 51.21) 0.3636 |
| High | 405.21 (349.65, 460.77) <0.0001 | 103.20 (62.77, 143.63) <0.0001 | 82.40 (46.34, 118.45) <0.0001 |
| Baseline FEF 25-75% (mL/s) |  |  |  |
| Iron,refigerated (umol/L) | 19.31 (14.02, 24.60) <0.0001 | 6.12 (1.65, 10.59) 0.0073 | 6.72 (2.30, 11.13) 0.0029 |
| Iron,refigerated (umol/L) Tertile |  |  |  |
| Low | 0 | 0 | 0 |
| Medium | 84.76 (2.34, 167.19) 0.0439 | 5.55 (-62.67, 73.78) 0.8732 | 8.10 (-58.71, 74.92) 0.8121 |
| High | 304.71 (222.18, 387.23) <0.0001 | 83.33 (13.62, 153.04) 0.0192 | 89.96 (21.12, 158.81) 0.0105 |
| ***Stratification by respiratory diease*** |  |  |  |
| Respiratory disease= Yes |  |  |  |
| Baseline FVC (mL) |  |  |  |
| Iron,refigerated (umol/L) | 26.70 (17.14, 36.27) <0.0001 | 7.35 (0.47, 14.24) 0.0365 | 5.12 (-0.72, 10.96) 0.0859 |
| Iron,refigerated (umol/L) Tertile |  |  |  |
| Low | 0 | 0 | 0 |
| Medium | 137.71 (-12.50, 287.93) 0.0726 | 50.48 (-56.19, 157.15) 0.3539 | 11.91 (-77.66, 101.48) 0.7944 |
| High | 394.68 (244.21, 545.14) <0.0001 | 130.61 (22.40, 238.83) 0.0182 | 76.32 (-16.11, 168.75) 0.1059 |
| Baseline FEV 1 (mL) |  |  |  |
| Iron,refigerated (umol/L) | 21.79 (13.59, 29.98) <0.0001 | 6.89 (1.02, 12.77) 0.0217 | 5.49 (0.33, 10.65) 0.0372 |
| Iron,refigerated (umol/L) Tertile |  |  |  |
| Low | 0 | 0 | 0 |
| Medium | 76.33 (-52.33, 204.99) 0.2452 | 47.95 (-43.11, 139.02) 0.3023 | 17.02 (-62.16, 96.21) 0.6735 |
| High | 320.34 (191.48, 449.21) <0.0001 | 118.27 (25.89, 210.65) 0.0123 | 84.02 (2.30, 165.73) 0.0441 |
| Baseline FEF 25-75% (mL/s) |  |  |  |
| Iron,refigerated (umol/L) | 18.11 (5.51, 30.71) 0.0049 | 5.60 (-4.61, 15.81) 0.2825 | 5.69 (-4.32, 15.70) 0.2656 |
| Iron,refigerated (umol/L) Tertile |  |  |  |
| Low | 0 | 0 | 0 |
| Medium | -2.21 (-199.81, 195.38) 0.9825 | 62.94 (-95.42, 221.30) 0.4362 | 40.78 (-112.85, 194.42) 0.6030 |
| High | 257.49 (59.57, 455.40) 0.0109 | 92.85 (-67.80, 253.49) 0.2576 | 87.33 (-71.21, 245.88) 0.2806 |
| Respiratory disease= No |  |  |  |
| Baseline FVC (mL) |  |  |  |
| Iron,refigerated (umol/L) | 35.47 (30.57, 40.37) <0.0001 | 6.58 (3.01, 10.15) 0.0003 | 5.10 (2.03, 8.17) 0.0012 |
| Iron,refigerated (umol/L) Tertile |  |  |  |
| Low | 0 | 0 | 0 |
| Medium | 306.45 (229.62, 383.28) <0.0001 | 40.32 (-14.37, 95.01) 0.1485 | 26.88 (-19.73, 73.48) 0.2584 |
| High | 550.37 (473.87, 626.87) <0.0001 | 95.77 (40.05, 151.50) 0.0008 | 76.57 (28.71, 124.42) 0.0017 |
| Baseline FEV 1 (mL) |  |  |  |
| Iron,refigerated (umol/L) | 27.84 (23.75, 31.93) <0.0001 | 6.11 (3.18, 9.04) <0.0001 | 5.47 (2.83, 8.11) <0.0001 |
| Iron,refigerated (umol/L) Tertile |  |  |  |
| Low | 0 | 0 | 0 |
| Medium | 210.91 (146.82, 275.00) <0.0001 | 21.06 (-23.82, 65.94) 0.3577 | 17.54 (-22.47, 57.55) 0.3902 |
| High | 431.23 (367.41, 495.05) <0.0001 | 84.90 (39.17, 130.63) 0.0003 | 76.73 (35.65, 117.81) 0.0003 |
| Baseline FEF 25-75% (mL/s) |  |  |  |
| Iron,refigerated (umol/L) | 21.77 (15.68, 27.86) <0.0001 | 5.33 (0.28, 10.37) 0.0385 | 6.67 (1.66, 11.68) 0.0091 |
| Iron,refigerated (umol/L) Tertile |  |  |  |
| Low | 0 | 0 | 0 |
| Medium | 104.25 (8.85, 199.64) 0.0323 | -14.72 (-91.99, 62.56) 0.7090 | 0.19 (-75.75, 76.13) 0.9962 |
| High | 336.14 (241.15, 431.12) <0.0001 | 62.63 (-16.11, 141.36) 0.1191 | 81.49 (3.52, 159.47) 0.0406 |
| Respiratory disease= Unknown |  |  |  |
| Baseline FVC (mL) |  |  |  |
| Iron,refigerated (umol/L) | 48.75 (23.89, 73.61) 0.0002 | 27.19 (9.69, 44.69) 0.0028 | 19.08 (1.10, 37.06) 0.0396 |
| Iron,refigerated (umol/L) Tertile |  |  |  |
| Low | 0 | 0 | 0 |
| Medium | 144.95 (-201.90, 491.80) 0.4140 | -7.25 (-244.58, 230.08) 0.9523 | -161.74 (-410.41, 86.92) 0.2047 |
| High | 749.70 (343.89, 1155.50) 0.0004 | 419.28 (139.29, 699.26) 0.0039 | 248.26 (-34.88, 531.40) 0.0882 |
| Baseline FEV 1 (mL) |  |  |  |
| Iron,refigerated (umol/L) | 38.50 (17.42, 59.57) 0.0005 | 24.82 (9.80, 39.83) 0.0015 | 18.82 (3.37, 34.27) 0.0184 |
| Iron,refigerated (umol/L) Tertile |  |  |  |
| Low | 0 | 0 | 0 |
| Medium | 123.99 (-168.63, 416.62) 0.4076 | 8.83 (-194.66, 212.33) 0.9323 | -141.29 (-353.85, 71.26) 0.1950 |
| High | 624.39 (282.04, 966.75) 0.0005 | 391.27 (151.19, 631.34) 0.0017 | 267.69 (25.66, 509.71) 0.0321 |
| Baseline FEF 25-75% (mL/s) |  |  |  |
| Iron,refigerated (umol/L) | 31.01 (-0.14, 62.16) 0.0529 | 26.95 (-0.21, 54.11) 0.0537 | 25.21 (-2.14, 52.55) 0.0732 |
| Iron,refigerated (umol/L) Tertile |  |  |  |
| Low | 0 | 0 | 0 |
| Medium | 73.71 (-355.91, 503.32) 0.7371 | -28.83 (-395.21, 337.55) 0.8776 | -189.59(-566.15, 186.97) 0.3257 |
| High | 620.50 (117.87, 1123.12) 0.0167 | 497.30 (65.07, 929.53) 0.0256 | 428.26 (-0.50, 857.03) 0.0525 |
| Total |  |  |  |
| Baseline FVC (mL) |  |  |  |
| Iron,refigerated (umol/L) | 34.04 (29.74, 38.33) <0.0001 | 7.27 (4.16, 10.39) <0.0001 | 5.45 (2.78, 8.13) <0.0001 |
| Iron,refigerated (umol/L) Tertile |  |  |  |
| Low | 0 | 0 | 0 |
| Medium | 266.50 (199.36, 333.63) <0.0001 | 41.68 (-5.93, 89.30) 0.0862 | 20.50 (-19.94, 60.94) 0.3204 |
| High | 521.07 (453.88, 588.27) <0.0001 | 109.25 (60.60, 157.91) <0.0001 | 79.32 (37.65, 120.99) 0.0002 |
| Baseline FEV 1 (mL) |  |  |  |
| Iron,refigerated (umol/L) | 26.89 (23.28, 30.49) <0.0001 | 6.81 (4.22, 9.39) <0.0001 | 5.80 (3.49, 8.11) <0.0001 |
| Iron,refigerated (umol/L) Tertile |  |  |  |
| Low | 0 | 0 | 0 |
| Medium | 180.64 (124.33, 236.95) <0.0001 | 26.56 (-12.91, 66.03) 0.1873 | 16.22 (-18.77, 51.21) 0.3636 |
| High | 411.55 (355.18, 467.91) <0.0001 | 98.87 (58.54, 139.20) <0.0001 | 82.40 (46.34, 118.45) <0.0001 |
| Baseline FEF 25-75% (mL/s) |  |  |  |
| Iron,refigerated (umol/L) | 21.27 (15.87, 26.67) <0.0001 | 6.03 (1.57, 10.49) 0.0081 | 6.72 (2.30, 11.13) 0.0029 |
| Iron,refigerated (umol/L) Tertile |  |  |  |
| Low | 0 | 0 | 0 |
| Medium | 81.92 (-2.40, 166.24) 0.0569 | 0.17 (-68.02, 68.36) 0.9961 | 8.10 (-58.71, 74.92) 0.8121 |
| High | 325.58 (241.18, 409.98) <0.0001 | 79.60 (9.92, 149.29) 0.0252 | 89.96 (21.12, 158.81) 0.0105 |
| ***Stratification by standing height*** |  |  |  |
| Standing Height (cm) Tertile= Low |  |  |  |
| Baseline FVC (mL) |  |  |  |
| Iron,refigerated (umol/L) | 13.57 (8.75, 18.40) <0.0001 | 5.52 (1.54, 9.51) 0.0067 | 3.87 (-0.03, 7.77) 0.0520 |
| Iron,refigerated (umol/L) Tertile |  |  |  |
| Low | 0 | 0 | 0 |
| Medium | 29.75 (-41.54, 101.05) 0.4135 | -8.84 (-66.86, 49.18) 0.7652 | -22.96 (-79.06, 33.13) 0.4224 |
| High | 209.53 (135.07, 283.98) <0.0001 | 70.25 (8.85, 131.64) 0.0250 | 50.21 (-9.63, 110.05) 0.1002 |
| Baseline FEV 1 (mL) |  |  |  |
| Iron,refigerated (umol/L) | 11.16 (6.73, 15.60) <0.0001 | 4.31 (0.95, 7.68) 0.0121 | 3.37 (0.02, 6.73) 0.0486 |
| Iron,refigerated (umol/L) Tertile |  |  |  |
| Low | 0 | 0 | 0 |
| Medium | 17.45 (-47.96, 82.86) 0.6012 | -9.61 (-58.61, 39.39) 0.7008 | -17.98 (-66.15, 30.19) 0.4646 |
| High | 189.19 (120.88, 257.51) <0.0001 | 63.10 (11.25, 114.95) 0.0172 | 51.63 (0.24, 103.02) 0.0491 |
| Baseline FEF 25-75% (mL/s) |  |  |  |
| Iron,refigerated (umol/L) | 7.98 (0.06, 15.91) 0.0485 | 1.42 (-5.16, 8.00) 0.6724 | 2.02 (-4.62, 8.66) 0.5512 |
| Iron,refigerated (umol/L) Tertile |  |  |  |
| Low | 0 | 0 | 0 |
| Medium | -0.03 (-116.94, 116.88) 0.9996 | -11.20 (-106.99, 84.59) 0.8188 | -7.23 (-102.75, 88.28) 0.8820 |
| High | 193.15 (71.06, 315.24) 0.0020 | 54.29 (-47.07, 155.65) 0.2939 | 63.15 (-38.74, 165.05) 0.2246 |
| Standing Height (cm) Tertile= Medium |  |  |  |
| Baseline FVC (mL) |  |  |  |
| Iron,refigerated (umol/L) | 23.06 (17.45, 28.67) <0.0001 | 6.52 (2.17, 10.86) 0.0033 | 5.05 (0.70, 9.40) 0.0229 |
| Iron,refigerated (umol/L) Tertile |  |  |  |
| Low | 0 | 0 | 0 |
| Medium | 174.10 (89.20, 259.00) <0.0001 | 67.64 (3.10, 132.18) 0.0401 | 57.06 (-6.07, 120.19) 0.0766 |
| High | 368.57 (282.58, 454.56) <0.0001 | 109.36 (42.45, 176.28) 0.0014 | 79.07 (12.40, 145.74) 0.0202 |
| Baseline FEV 1 (mL) |  |  |  |
| Iron,refigerated (umol/L) | 17.46 (12.28, 22.63) <0.0001 | 5.43 (1.70, 9.15) 0.0043 | 4.76 (0.97, 8.55) 0.0138 |
| Iron,refigerated (umol/L) Tertile |  |  |  |
| Low | 0 | 0 | 0 |
| Medium | 109.42 (31.15, 187.69) 0.0062 | 58.61 (3.29, 113.93) 0.0380 | 51.88 (-3.12, 106.87) 0.0647 |
| High | 284.22 (204.95, 363.49) <0.0001 | 94.51 (37.16, 151.86) 0.0013 | 78.01 (19.93, 136.10) 0.0086 |
| Baseline FEF 25-75% (mL/s) |  |  |  |
| Iron,refigerated (umol/L) | 11.40 (2.34, 20.47) 0.0137 | 2.01 (-5.36, 9.38) 0.5924 | 3.01 (-4.52, 10.54) 0.4339 |
| Iron,refigerated (umol/L) Tertile |  |  |  |
| Low | 0 | 0 | 0 |
| Medium | -1.29 (-138.46, 135.89) 0.9853 | 20.83 (-88.75, 130.40) 0.7095 | 23.03 (-86.37, 132.42) 0.6800 |
| High | 200.78 (61.85, 339.72) 0.0047 | 50.79 (-62.81, 164.39) 0.3810 | 62.36 (-53.17, 177.90) 0.2902 |
| Standing Height (cm) Tertile= High |  |  |  |
| Baseline FVC (mL) |  |  |  |
| Iron,refigerated (umol/L) | 19.32 (12.81, 25.84) <0.0001 | 7.31 (1.96, 12.65) 0.0075 | 6.88 (1.54, 12.23) 0.0116 |
| Iron,refigerated (umol/L) Tertile |  |  |  |
| Low | 0 | 0 | 0 |
| Medium | 162.34 (52.45, 272.22) 0.0038 | 42.41 (-46.98, 131.80) 0.3525 | 29.70 (-59.30, 118.70) 0.5132 |
| High | 312.03 (205.72, 418.34) <0.0001 | 110.47 (23.17, 197.77) 0.0132 | 100.83 (13.67, 187.98) 0.0235 |
| Baseline FEV 1 (mL) |  |  |  |
| Iron,refigerated (umol/L) | 18.75 (12.82, 24.68) <0.0001 | 8.87 (4.30, 13.43) 0.0001 | 8.58 (4.00, 13.15) 0.0002 |
| Iron,refigerated (umol/L) Tertile |  |  |  |
| Low | 0 | 0 | 0 |
| Medium | 104.39 (4.32, 204.45) 0.0410 | 16.17 (-60.15, 92.49) 0.6780 | 17.47 (-58.76, 93.70) 0.6534 |
| High | 276.72 (179.90, 373.53) <0.0001 | 113.03 (38.50, 187.57) 0.0030 | 111.59 (36.94, 186.24) 0.0034 |
| Baseline FEF 25-75% (mL/s) |  |  |  |
| Iron,refigerated (umol/L) | 22.20 (11.92, 32.48) <0.0001 | 13.26 (4.80, 21.72) 0.0022 | 13.99 (5.49, 22.48) 0.0013 |
| Iron,refigerated (umol/L) Tertile |  |  |  |
| Low | 0 | 0 | 0 |
| Medium | 46.87 (-126.78, 220.52) 0.5969 | -12.02 (-153.61, 129.57) 0.8679 | 12.74 (-128.91, 154.40) 0.8600 |
| High | 265.90 (97.90, 433.89) 0.0020 | 121.09 (-17.19, 259.38) 0.0863 | 140.65 (1.93, 279.36) 0.0471 |
| Total |  |  |  |
| Baseline FVC (mL) |  |  |  |
| Iron,refigerated (umol/L) | 18.64 (15.35, 21.94) <0.0001 | 6.40 (3.71, 9.10) <0.0001 | 5.45 (2.78, 8.13) <0.0001 |
| Iron,refigerated (umol/L) Tertile |  |  |  |
| Low | 0 | 0 | 0 |
| Medium | 116.81 (65.69, 167.93) <0.0001 | 32.43 (-8.82, 73.68) 0.1234 | 20.50 (-19.94, 60.94) 0.3204 |
| High | 291.95 (240.52, 343.38) <0.0001 | 96.30 (54.17, 138.44) <0.0001 | 79.32 (37.65, 120.99) 0.0002 |
| Baseline FEV 1 (mL) |  |  |  |
| Iron,refigerated (umol/L) | 15.83 (12.81, 18.85) <0.0001 | 6.23 (3.93, 8.54) <0.0001 | 5.80 (3.49, 8.11) <0.0001 |
| Iron,refigerated (umol/L) Tertile |  |  |  |
| Low | 0 | 0 | 0 |
| Medium | 72.97 (26.18, 119.76) 0.0022 | 20.60 (-14.63, 55.83) 0.2518 | 16.22 (-18.77, 51.21) 0.3636 |
| High | 247.23 (200.15, 294.31) <0.0001 | 90.29 (54.30, 126.27) <0.0001 | 82.40 (46.34, 118.45) <0.0001 |
| Baseline FEF 25-75% (mL/s) |  |  |  |
| Iron,refigerated (umol/L) | 14.02 (8.73, 19.31) <0.0001 | 5.76 (1.37, 10.14) 0.0101 | 6.72 (2.30, 11.13) 0.0029 |
| Iron,refigerated (umol/L) Tertile |  |  |  |
| Low | 0 | 0 | 0 |
| Medium | 11.82 (-70.18, 93.83) 0.7775 | -1.42 (-68.41, 65.56) 0.9668 | 8.10 (-58.71, 74.92) 0.8121 |
| High | 218.67 (136.16, 301.18) <0.0001 | 75.65 (7.24, 144.07) 0.0303 | 89.96 (21.12, 158.81) 0.0105 |
| ***Stratification by BMI*** |  |  |  |
| Body Mass Index (kg/m**2)= < 25 |  |  |  |
| Baseline FVC (mL) |  |  |  |
| Iron,refigerated (umol/L) | 20.74 (14.39, 27.09) <0.0001 | 6.42 (1.37, 11.46) 0.0127 | 3.38 (-0.71, 7.47) 0.1051 |
| Iron,refigerated (umol/L) Tertile |  |  |  |
| Low | 0 | 0 | 0 |
| Medium | 202.03 (90.15, 313.92) 0.0004 | 63.92 (-23.54, 151.38) 0.1522 | 30.38 (-40.25, 101.00) 0.3994 |
| High | 322.20 (215.70, 428.71) <0.0001 | 101.38 (17.08, 185.67) 0.0185 | 50.22 (-18.13, 118.56) 0.1500 |
| Baseline FEV 1 (mL) |  |  |  |
| Iron,refigerated (umol/L) | 18.09 (12.68, 23.51) <0.0001 | 6.59 (2.35, 10.83) 0.0023 | 4.61 (1.01, 8.21) 0.0121 |
| Iron,refigerated (umol/L) Tertile |  |  |  |
| Low | 0 | 0 | 0 |
| Medium | 145.20 (49.75, 240.64) 0.0029 | 46.22 (-27.25, 119.69) 0.2177 | 25.82 (-36.34, 87.97) 0.4157 |
| High | 274.68 (183.82, 365.55) <0.0001 | 96.71 (25.90, 167.51) 0.0075 | 65.10 (4.95, 125.25) 0.0340 |
| Baseline FEF 25-75% (mL/s) |  |  |  |
| Iron,refigerated (umol/L) | 17.63 (9.18, 26.09) <0.0001 | 7.44 (0.52, 14.36) 0.0353 | 6.70 (0.02, 13.39) 0.0496 |
| Iron,refigerated (umol/L) Tertile |  |  |  |
| Low | 0 | 0 | 0 |
| Medium | 90.75 (-58.14, 239.64) 0.2324 | 28.29 (-91.78, 148.35) 0.6443 | 20.66 (-94.81, 136.13) 0.7259 |
| High | 256.02 (114.29, 397.76) 0.0004 | 97.36 (-18.35, 213.08) 0.0993 | 89.22 (-22.52, 200.96) 0.1178 |
| Body Mass Index (kg/m**2)= >= 25 |  |  |  |
| Baseline FVC (mL) |  |  |  |
| Iron,refigerated (umol/L) | 45.18 (39.30, 51.05) <0.0001 | 10.73 (6.79, 14.68) <0.0001 | 8.47 (4.98, 11.96) <0.0001 |
| Iron,refigerated (umol/L) Tertile |  |  |  |
| Low | 0 | 0 | 0 |
| Medium | 306.83 (221.90, 391.76) <0.0001 | 45.94 (-9.31, 101.18) 0.1033 | 25.76 (-22.85, 74.36) 0.2991 |
| High | 670.91 (582.76, 759.06) <0.0001 | 151.51 (92.41, 210.61) <0.0001 | 114.28 (62.08, 166.48) <0.0001 |
| Baseline FEV 1 (mL) |  |  |  |
| Iron,refigerated (umol/L) | 32.92 (28.04, 37.81) <0.0001 | 8.55 (5.23, 11.87) <0.0001 | 7.33 (4.31, 10.35) <0.0001 |
| Iron,refigerated (umol/L) Tertile |  |  |  |
| Low | 0 | 0 | 0 |
| Medium | 200.33 (129.75, 270.90) <0.0001 | 26.53 (-19.94, 73.00) 0.2632 | 15.70 (-26.41, 57.81) 0.4650 |
| High | 498.36 (425.11, 571.61) <0.0001 | 122.97 (73.26, 172.68) <0.0001 | 100.70 (55.48, 145.93) <0.0001 |
| Baseline FEF 25-75% (mL/s) |  |  |  |
| Iron,refigerated (umol/L) | 21.52 (14.43, 28.61) <0.0001 | 6.56 (0.62, 12.50) 0.0305 | 6.48 (0.60, 12.36) 0.0307 |
| Iron,refigerated (umol/L) Tertile |  |  |  |
| Low | 0 | 0 | 0 |
| Medium | 74.05 (-28.26, 176.36) 0.1561 | -2.50 (-85.65, 80.64) 0.9529 | -2.53 (-84.46, 79.39) 0.9517 |
| High | 345.61 (239.42, 451.81) <0.0001 | 93.83 (4.90, 182.77) 0.0387 | 87.42 (-0.56, 175.40) 0.0516 |
| Total |  |  |  |
| Baseline FVC (mL) |  |  |  |
| Iron,refigerated (umol/L) | 35.17 (30.80, 39.54) <0.0001 | 8.68 (5.55, 11.82) <0.0001 | 5.45 (2.78, 8.13) <0.0001 |
| Iron,refigerated (umol/L) Tertile |  |  |  |
| Low | 0 | 0 | 0 |
| Medium | 278.18 (210.35, 346.00) <0.0001 | 52.41 (4.78, 100.04) 0.0311 | 20.50 (-19.94, 60.94) 0.3204 |
| High | 542.52 (474.33, 610.71) <0.0001 | 131.18 (82.29, 180.08) <0.0001 | 79.32 (37.65, 120.99) 0.0002 |
| Baseline FEV 1 (mL) |  |  |  |
| Iron,refigerated (umol/L) | 26.85 (23.19, 30.51) <0.0001 | 7.69 (5.08, 10.30) <0.0001 | 5.80 (3.49, 8.11) <0.0001 |
| Iron,refigerated (umol/L) Tertile |  |  |  |
| Low | 0 | 0 | 0 |
| Medium | 185.90 (129.12, 242.68) <0.0001 | 34.29 (-5.30, 73.89) 0.0896 | 16.22 (-18.77, 51.21) 0.3636 |
| High | 415.48 (358.40, 472.56) <0.0001 | 113.00 (72.36, 153.65) <0.0001 | 82.40 (46.34, 118.45) <0.0001 |
| Baseline FEF 25-75% (mL/s) |  |  |  |
| Iron,refigerated (umol/L) | 19.93 (14.50, 25.36) <0.0001 | 7.10 (2.60, 11.60) 0.0020 | 6.72 (2.30, 11.13) 0.0029 |
| Iron,refigerated (umol/L) Tertile |  |  |  |
| Low | 0 | 0 | 0 |
| Medium | 80.97 (-3.30, 165.24) 0.0597 | 9.85 (-58.50, 78.19) 0.7776 | 8.10 (-58.71, 74.92) 0.8121 |
| High | 310.72 (226.00, 395.44) <0.0001 | 95.80 (25.65, 165.95) 0.0075 | 89.96 (21.12, 158.81) 0.0105 |
| Baseline FVC (mL) |  |  |  |
| Iron,refigerated (umol/L) | 33.64 (28.97, 38.30) <0.0001 | 7.42 (3.96, 10.87) <0.0001 | 5.59 (2.67, 8.51) 0.0002 |
| Iron,refigerated (umol/L) Tertile |  |  |  |
| Low | 0 | 0 | 0 |
| Medium | 297.37 (223.24, 371.51) <0.0001 | 50.60 (-3.19, 104.40) 0.0653 | 20.68 (-24.33, 65.69) 0.3679 |
| High | 522.31 (449.11, 595.51) <0.0001 | 115.98 (61.83, 170.14) <0.0001 | 85.70 (40.04, 131.37) 0.0002 |
| Baseline FEV 1 (mL) |  |  |  |
| Iron,refigerated (umol/L) | 26.48 (22.61, 30.36) <0.0001 | 6.84 (4.00, 9.68) <0.0001 | 5.56 (3.05, 8.07) <0.0001 |
| Iron,refigerated (umol/L) Tertile |  |  |  |
| Low | 0 | 0 | 0 |
| Medium | 210.87 (149.22, 272.51) <0.0001 | 37.85 (-6.42, 82.11) 0.0938 | 17.20 (-21.50, 55.89) 0.3838 |
| High | 410.86 (350.00, 471.73) <0.0001 | 102.01 (57.45, 146.57) <0.0001 | 80.57 (41.31, 119.83) <0.0001 |
| Baseline FEF 25-75% (mL/s) |  |  |  |
| Iron,refigerated (umol/L) | 20.16 (14.40, 25.91) <0.0001 | 5.45 (0.61, 10.28) 0.0273 | 5.32 (0.55, 10.09) 0.0290 |
| Iron,refigerated (umol/L) Tertile |  |  |  |
| Low | 0 | 0 | 0 |
| Medium | 108.94 (17.51, 200.38) 0.0196 | 7.58 (-67.70, 82.86) 0.8436 | -0.56 (-74.08, 72.97) 0.9881 |
| High | 319.92 (229.64, 410.20) <0.0001 | 78.13 (2.35, 153.92) 0.0434 | 74.06 (-0.53, 148.65) 0.0517 |
| ***Stratification by Systolic Blood Pressure*** | |  |  |
| Systolic Blood Pressure (mm Hg)= >= 140 | |  |  |
| Baseline FVC (mL) |  |  |  |
| Iron,refigerated (umol/L) | 41.94 (28.39, 55.48) <0.0001 | 6.10 (-3.05, 15.25) 0.1915 | 5.37 (-3.17, 13.90) 0.2183 |
| Iron,refigerated (umol/L) Tertile |  |  |  |
| Low | 0 | 0 | 0 |
| Medium | 276.36 (82.24, 470.48) 0.0054 | 56.62 (-70.65, 183.89) 0.3836 | 59.57 (-58.40, 177.54) 0.3227 |
| High | 642.44 (438.68, 846.21) <0.0001 | 103.91 (-34.46, 242.27) 0.1416 | 72.20 (-56.55, 200.95) 0.2722 |
| Baseline FEV 1 (mL) |  |  |  |
| Iron,refigerated (umol/L) | 31.49 (20.75, 42.23) <0.0001 | 6.75 (-1.12, 14.62) 0.0934 | 6.66 (-0.83, 14.15) 0.0821 |
| Iron,refigerated (umol/L) Tertile |  |  |  |
| Low | 0 | 0 | 0 |
| Medium | 153.63 (-0.05, 307.30) 0.0505 | 14.72 (-94.72, 124.16) 0.7922 | 37.03 (-66.50, 140.55) 0.4836 |
| High | 487.15 (325.84, 648.46) <0.0001 | 112.95 (-6.02, 231.93) 0.0633 | 104.36 (-8.62, 217.35) 0.0708 |
| Baseline FEF 25-75% (mL/s) |  |  |  |
| Iron,refigerated (umol/L) | 25.88 (9.85, 41.92) 0.0016 | 11.23 (-3.43, 25.89) 0.1337 | 13.76 (-0.83, 28.36) 0.0650 |
| Iron,refigerated (umol/L) Tertile |  |  |  |
| Low | 0 | 0 | 0 |
| Medium | 13.92 (-215.96, 243.79) 0.9056 | -29.01 (-232.98, 174.95) 0.7805 | 40.42 (-161.38, 242.22) 0.6948 |
| High | 361.23 (119.94, 602.52) 0.0035 | 128.04 (-93.70, 349.78) 0.2582 | 174.46 (-45.78, 394.70) 0.1211 |
| Systolic Blood Pressure (mm Hg)= Unknown | |  |  |
| Baseline FVC (mL) |  |  |  |
| Iron,refigerated (umol/L) | 32.93 (15.52, 50.35) 0.0002 | 8.71 (-3.01, 20.42) 0.1461 |  |
| Iron,refigerated (umol/L) Tertile |  |  |  |
| Low | 0 | 0 |  |
| Medium | 106.75 (-144.01, 357.52) 0.4047 | -27.00 (-193.29, 139.29) 0.7505 |  |
| High | 446.53 (169.33, 723.74) 0.0017 | 76.86 (-109.15, 262.87) 0.4186 |  |
| Baseline FEV 1 (mL) |  |  |  |
| Iron,refigerated (umol/L) | 29.28 (13.99, 44.58) 0.0002 | 10.16 (-0.24, 20.56) 0.0565 |  |
| Iron,refigerated (umol/L) Tertile |  |  |  |
| Low | 0 | 0 |  |
| Medium | 75.81 (-144.19, 295.81) 0.4999 | -16.87 (-164.42, 130.68) 0.8228 |  |
| High | 403.19 (159.99, 646.38) 0.0013 | 123.37 (-41.68, 288.42) 0.1439 |  |
| Baseline FEF 25-75% (mL/s) |  |  |  |
| Iron,refigerated (umol/L) | 30.48 (7.26, 53.71) 0.0105 | 14.21 (-4.84, 33.26) 0.1447 |  |
| Iron,refigerated (umol/L) Tertile |  |  |  |
| Low | 0 | 0 |  |
| Medium | 115.06 (-218.96, 449.08) 0.5000 | 72.95 (-197.43, 343.32) 0.5973 |  |
| High | 382.52 (13.29, 751.76) 0.0431 | 185.85 (-116.58, 488.29) 0.2293 |  |
| Total |  |  |  |
| Baseline FVC (mL) |  |  |  |
| Iron,refigerated (umol/L) | 34.43 (30.16, 38.71) <0.0001 | 7.49 (4.37, 10.61) <0.0001 | 5.45 (2.78, 8.13) <0.0001 |
| Iron,refigerated (umol/L) Tertile |  |  |  |
| Low | 0 | 0 | 0 |
| Medium | 281.73 (214.96, 348.51) <0.0001 | 45.99 (-1.71, 93.68) 0.0588 | 20.50 (-19.94, 60.94) 0.3204 |
| High | 529.43 (462.61, 596.25) <0.0001 | 114.15 (65.42, 162.88) <0.0001 | 79.32 (37.65, 120.99) 0.0002 |
| Baseline FEV 1 (mL) |  |  |  |
| Iron,refigerated (umol/L) | 27.15 (23.60, 30.69) <0.0001 | 7.07 (4.48, 9.66) <0.0001 | 5.80 (3.49, 8.11) <0.0001 |
| Iron,refigerated (umol/L) Tertile |  |  |  |
| Low | 0 | 0 | 0 |
| Medium | 194.73 (139.32, 250.14) <0.0001 | 30.97 (-8.59, 70.53) 0.1249 | 16.22 (-18.77, 51.21) 0.3636 |
| High | 417.15 (361.70, 472.59) <0.0001 | 104.27 (63.86, 144.69) <0.0001 | 82.40 (46.34, 118.45) <0.0001 |
| Baseline FEF 25-75% (mL/s) |  |  |  |
| Iron,refigerated (umol/L) | 21.32 (16.04, 26.60) <0.0001 | 6.44 (1.97, 10.91) 0.0047 | 6.72 (2.30, 11.13) 0.0029 |
| Iron,refigerated (umol/L) Tertile |  |  |  |
| Low | 0 | 0 | 0 |
| Medium | 97.88 (15.52, 180.24) 0.0199 | 5.59 (-62.66, 73.85) 0.8724 | 8.10 (-58.71, 74.92) 0.8121 |
| High | 327.09 (244.68, 409.50) <0.0001 | 87.01 (17.27, 156.75) 0.0145 | 89.96 (21.12, 158.81) 0.0105 |
| ***Stratification by Diastolic Blood Pressure*** |  |  |  |
| Diastolic Blood Pressure (mm Hg)= <90 |  |  |  |
| Baseline FVC (mL) |  |  |  |
| Iron,refigerated (umol/L) | 34.86 (30.25, 39.46) <0.0001 | 7.37 (4.03, 10.72) <0.0001 | 5.68 (2.83, 8.53) <0.0001 |
| Iron,refigerated (umol/L) Tertile |  |  |  |
| Low | 0 | 0 | 0 |
| Medium | 284.27 (212.26, 356.29) <0.0001 | 44.44 (-6.72, 95.60) 0.0887 | 18.41 (-24.72, 61.54) 0.4029 |
| High | 538.06 (466.58, 609.55) <0.0001 | 115.01 (63.15, 166.86) <0.0001 | 83.76 (39.66, 127.86) 0.0002 |
| Baseline FEV 1 (mL) |  |  |  |
| Iron,refigerated (umol/L) | 27.58 (23.71, 31.44) <0.0001 | 6.79 (4.02, 9.55) <0.0001 | 5.70 (3.24, 8.15) <0.0001 |
| Iron,refigerated (umol/L) Tertile |  |  |  |
| Low | 0 | 0 | 0 |
| Medium | 197.02 (136.59, 257.45) <0.0001 | 30.40 (-11.94, 72.74) 0.1594 | 14.28 (-22.95, 51.50) 0.4523 |
| High | 424.89 (364.91, 484.87) <0.0001 | 100.21 (57.29, 143.13) <0.0001 | 79.74 (41.67, 117.80) <0.0001 |
| Baseline FEF 25-75% (mL/s) |  |  |  |
| Iron,refigerated (umol/L) | 21.75 (16.00, 27.49) <0.0001 | 5.65 (0.93, 10.37) 0.0190 | 5.90 (1.25, 10.56) 0.0129 |
| Iron,refigerated (umol/L) Tertile |  |  |  |
| Low | 0 | 0 | 0 |
| Medium | 92.95 (3.20, 182.70) 0.0424 | -1.13 (-73.37, 71.12) 0.9756 | -2.16 (-72.65, 68.32) 0.9520 |
| High | 335.76 (246.68, 424.85) <0.0001 | 72.84 (-0.38, 146.06) 0.0513 | 73.95 (1.89, 146.02) 0.0443 |
| Diastolic Blood Pressure (mm Hg)= >= 90 | |  |  |
| Baseline FVC (mL) |  |  |  |
| Iron,refigerated (umol/L) | 38.13 (19.13, 57.13) 0.0001 | 7.22 (-6.00, 20.45) 0.2854 | 3.84 (-8.35, 16.03) 0.5374 |
| Iron,refigerated (umol/L) Tertile |  |  |  |
| Low | 0 | 0 | 0 |
| Medium | 408.03 (86.41, 729.64) 0.0136 | 203.27 (-12.19, 418.72) 0.0657 | 154.88 (-40.87, 350.63) 0.1224 |
| High | 693.66 (363.94, 1023.37) <0.0001 | 161.14 (-65.94, 388.21) 0.1655 | 73.78 (-134.05, 281.62) 0.4873 |
| Baseline FEV 1 (mL) |  |  |  |
| Iron,refigerated (umol/L) | 28.60 (13.36, 43.85) 0.0003 | 8.74 (-2.10, 19.57) 0.1154 | 5.87 (-4.52, 16.26) 0.2694 |
| Iron,refigerated (umol/L) Tertile |  |  |  |
| Low | 0 | 0 | 0 |
| Medium | 251.85 (-5.89, 509.58) 0.0566 | 137.21 (-39.71, 314.14) 0.1298 | 104.29 (-63.03, 271.62) 0.2231 |
| High | 536.33 (272.10, 800.55) <0.0001 | 180.76 (-5.71, 367.22) 0.0586 | 117.67 (-59.99, 295.33) 0.1956 |
| Baseline FEF 25-75% (mL/s) |  |  |  |
| Iron,refigerated (umol/L) | 21.39 (-1.52, 44.30) 0.0684 | 12.34 (-8.74, 33.43) 0.2522 | 8.34 (-13.66, 30.35) 0.4582 |
| Iron,refigerated (umol/L) Tertile |  |  |  |
| Low | 0 | 0 | 0 |
| Medium | 72.85 (-314.41, 460.12) 0.7126 | 63.51 (-281.15, 408.17) 0.7183 | 50.28 (-304.41, 404.96) 0.7814 |
| High | 446.57 (49.55, 843.58) 0.0284 | 251.24 (-112.01, 614.49) 0.1765 | 185.73 (-190.86, 562.31) 0.3348 |
| Diastolic Blood Pressure (mm Hg)= Unknown | |  |  |
| Baseline FVC (mL) |  |  |  |
| Iron,refigerated (umol/L) | 32.93 (15.52, 50.35) 0.0002 | 8.71 (-3.01, 20.42) 0.1461 |  |
| Iron,refigerated (umol/L) Tertile |  |  |  |
| Low | 0 | 0 |  |
| Medium | 106.75 (-144.01, 357.52) 0.4047 | -27.00 (-193.29, 139.29) 0.7505 |  |
| High | 446.53 (169.33, 723.74) 0.0017 | 76.86 (-109.15, 262.87) 0.4186 |  |
| Baseline FEV 1 (mL) |  |  |  |
| Iron,refigerated (umol/L) | 29.28 (13.99, 44.58) 0.0002 | 10.16 (-0.24, 20.56) 0.0565 |  |
| Iron,refigerated (umol/L) Tertile |  |  |  |
| Low | 0 | 0 |  |
| Medium | 75.81 (-144.19, 295.81) 0.4999 | -16.87 (-164.42, 130.68) 0.8228 |  |
| High | 403.19 (159.99, 646.38) 0.0013 | 123.37 (-41.68, 288.42) 0.1439 |  |
| Baseline FEF 25-75% (mL/s) |  |  |  |
| Iron,refigerated (umol/L) | 30.48 (7.26, 53.71) 0.0105 | 14.21 (-4.84, 33.26) 0.1447 |  |
| Iron,refigerated (umol/L) Tertile |  |  |  |
| Low | 0 | 0 |  |
| Medium | 115.06 (-218.96, 449.08) 0.5000 | 72.95 (-197.43, 343.32) 0.5973 |  |
| High | 382.52 (13.29, 751.76) 0.0431 | 185.85 (-116.58, 488.29) 0.2293 |  |
| Total |  |  |  |
| Baseline FVC (mL) |  |  |  |
| Iron,refigerated (umol/L) | 34.93 (30.60, 39.27) <0.0001 | 7.49 (4.36, 10.61) <0.0001 | 5.45 (2.78, 8.13) <0.0001 |
| Iron,refigerated (umol/L) Tertile |  |  |  |
| Low | 0 | 0 | 0 |
| Medium | 278.02 (210.32, 345.72) <0.0001 | 45.55 (-2.18, 93.28) 0.0615 | 20.50 (-19.94, 60.94) 0.3204 |
| High | 538.96 (471.23, 606.68) <0.0001 | 114.37 (65.60, 163.14) <0.0001 | 79.32 (37.65, 120.99) 0.0002 |
| Baseline FEV 1 (mL) |  |  |  |
| Iron,refigerated (umol/L) | 27.73 (24.09, 31.37) <0.0001 | 7.07 (4.48, 9.66) <0.0001 | 5.80 (3.49, 8.11) <0.0001 |
| Iron,refigerated (umol/L) Tertile |  |  |  |
| Low | 0 | 0 | 0 |
| Medium | 191.30 (134.47, 248.12) <0.0001 | 30.71 (-8.90, 70.31) 0.1287 | 16.22 (-18.77, 51.21) 0.3636 |
| High | 427.88 (371.04, 484.72) <0.0001 | 104.42 (63.95, 144.88) <0.0001 | 82.40 (46.34, 118.45) <0.0001 |
| Baseline FEF 25-75% (mL/s) |  |  |  |
| Iron,refigerated (umol/L) | 22.22 (16.81, 27.64) <0.0001 | 6.43 (1.96, 10.90) 0.0048 | 6.72 (2.30, 11.13) 0.0029 |
| Iron,refigerated (umol/L) Tertile |  |  |  |
| Low | 0 | 0 | 0 |
| Medium | 93.26 (8.72, 177.80) 0.0307 | 5.31 (-62.99, 73.61) 0.8789 | 8.10 (-58.71, 74.92) 0.8121 |
| High | 343.45 (258.88, 428.02) <0.0001 | 87.17 (17.39, 156.95) 0.0144 | 89.96 (21.12, 158.81) 0.0105 |
| ***Stratification by alcohol*** |  |  |  |
| Alcohol= Yes |  |  |  |
| Baseline FVC (mL) |  |  |  |
| Iron,refigerated (umol/L) | 32.37 (26.60, 38.14) <0.0001 | 6.57 (2.62, 10.52) 0.0011 | 5.52 (1.95, 9.08) 0.0024 |
| Iron,refigerated (umol/L) Tertile |  |  |  |
| Low | 0 | 0 | 0 |
| Medium | 330.24 (237.16, 423.32) <0.0001 | 84.91 (22.24, 147.58) 0.0080 | 49.99 (-6.26, 106.25) 0.0816 |
| High | 525.63 (434.05, 617.22) <0.0001 | 113.89 (51.15, 176.64) 0.0004 | 89.68 (33.01, 146.36) 0.0019 |
| Baseline FEV 1 (mL) |  |  |  |
| Iron,refigerated (umol/L) | 25.80 (21.01, 30.59) <0.0001 | 6.77 (3.49, 10.04) <0.0001 | 5.98 (2.96, 9.01) 0.0001 |
| Iron,refigerated (umol/L) Tertile |  |  |  |
| Low | 0 | 0 | 0 |
| Medium | 234.31 (156.96, 311.66) <0.0001 | 67.18 (15.13, 119.23) 0.0115 | 44.35 (-3.37, 92.07) 0.0686 |
| High | 416.04 (339.94, 492.15) <0.0001 | 108.94 (56.83, 161.06) <0.0001 | 91.84 (43.76, 139.92) 0.0002 |
| Baseline FEF 25-75% (mL/s) |  |  |  |
| Iron,refigerated (umol/L) | 21.69 (14.61, 28.77) <0.0001 | 7.70 (1.87, 13.53) 0.0097 | 7.62 (1.82, 13.42) 0.0100 |
| Iron,refigerated (umol/L) Tertile |  |  |  |
| Low | 0 | 0 | 0 |
| Medium | 150.34 (35.93, 264.74) 0.0101 | 62.61 (-30.06, 155.28) 0.1855 | 58.59 (-32.97, 150.14) 0.2098 |
| High | 342.54 (229.97, 455.12) <0.0001 | 109.00 (16.22, 201.78) 0.0214 | 108.40 (16.16, 200.64) 0.0213 |
| Alcohol= No |  |  |  |
| Baseline FVC (mL) |  |  |  |
| Iron,refigerated (umol/L) | 35.93 (24.63, 47.24) <0.0001 | 5.63 (-1.81, 13.06) 0.1382 | 4.14 (-2.72, 11.01) 0.2370 |
| Iron,refigerated (umol/L) Tertile |  |  |  |
| Low | 0 | 0 | 0 |
| Medium | 160.17 (16.32, 304.01) 0.0293 | -30.52 (-122.87, 61.84) 0.5174 | -24.39 (-108.68, 59.90) 0.5707 |
| High | 547.71 (386.78, 708.64) <0.0001 | 89.82 (-16.65, 196.30) 0.0986 | 67.50 (-30.98, 165.99) 0.1795 |
| Baseline FEV 1 (mL) |  |  |  |
| Iron,refigerated (umol/L) | 27.79 (18.28, 37.31) <0.0001 | 4.76 (-1.67, 11.19) 0.1472 | 3.44 (-2.60, 9.48) 0.2646 |
| Iron,refigerated (umol/L) Tertile |  |  |  |
| Low | 0 | 0 | 0 |
| Medium | 103.74 (-17.04, 224.52) 0.0926 | -33.95 (-113.79, 45.89) 0.4048 | -28.34 (-102.43, 45.74) 0.4535 |
| High | 446.22 (311.10, 581.35) <0.0001 | 93.10 (1.05, 185.14) 0.0477 | 74.34 (-12.22, 160.90) 0.0926 |
| Baseline FEF 25-75% (mL/s) |  |  |  |
| Iron,refigerated (umol/L) | 22.69 (8.79, 36.60) 0.0014 | 4.17 (-7.56, 15.89) 0.4864 | 2.70 (-9.03, 14.42) 0.6524 |
| Iron,refigerated (umol/L) Tertile |  |  |  |
| Low | 0 | 0 | 0 |
| Medium | 7.46 (-169.14, 184.07) 0.9340 | -86.63 (-232.22, 58.96) 0.2438 | -76.49 (-220.28, 67.29) 0.2974 |
| High | 416.14 (218.56, 613.72) <0.0001 | 115.36 (-52.48, 283.21) 0.1783 | 96.89 (-71.11, 264.89) 0.2586 |
| Alcohol= Unknown |  |  |  |
| Baseline FVC (mL) |  |  |  |
| Iron,refigerated (umol/L) | 25.14 (17.64, 32.65) <0.0001 | 6.91 (0.65, 13.17) 0.0307 | 6.46 (1.60, 11.33) 0.0092 |
| Iron,refigerated (umol/L) Tertile |  |  |  |
| Low | 0 | 0 | 0 |
| Medium | 156.96 (34.69, 279.22) 0.0120 | -10.75 (-110.89, 89.38) 0.8334 | 24.78 (-51.58, 101.14) 0.5249 |
| High | 367.66 (247.70, 487.61) <0.0001 | 82.81 (-16.96, 182.59) 0.1040 | 87.45 (10.29, 164.62) 0.0265 |
| Baseline FEV 1 (mL) |  |  |  |
| Iron,refigerated (umol/L) | 21.65 (15.21, 28.08) <0.0001 | 7.19 (1.81, 12.57) 0.0089 | 6.00 (1.62, 10.38) 0.0073 |
| Iron,refigerated (umol/L) Tertile |  |  |  |
| Low | 0 | 0 | 0 |
| Medium | 119.48 (14.68, 224.28) 0.0256 | -7.40 (-93.40, 78.61) 0.8662 | 19.65 (-49.16, 88.47) 0.5757 |
| High | 313.67 (210.85, 416.49) <0.0001 | 88.01 (2.32, 173.71) 0.0443 | 78.86 (9.31, 148.40) 0.0264 |
| Baseline FEF 25-75% (mL/s) |  |  |  |
| Iron,refigerated (umol/L) | 18.08 (8.44, 27.72) 0.0002 | 5.93 (-2.81, 14.66) 0.1837 | 4.22 (-4.19, 12.62) 0.3261 |
| Iron,refigerated (umol/L) Tertile |  |  |  |
| Low | 0 | 0 | 0 |
| Medium | 67.29 (-89.52, 224.09) 0.4005 | -23.27 (-162.84, 116.30) 0.7439 | 8.11 (-123.95, 140.16) 0.9043 |
| High | 256.21 (102.37, 410.06) 0.0011 | 67.31 (-71.76, 206.38) 0.3430 | 50.53 (-82.92, 183.98) 0.4581 |
| Total |  |  |  |
| Baseline FVC (mL) |  |  |  |
| Iron,refigerated (umol/L) | 30.80 (26.55, 35.06) <0.0001 | 6.61 (3.54, 9.69) <0.0001 | 5.45 (2.78, 8.13) <0.0001 |
| Iron,refigerated (umol/L) Tertile |  |  |  |
| Low | 0 | 0 | 0 |
| Medium | 250.40 (184.33, 316.48) <0.0001 | 37.37 (-9.51, 84.25) 0.1183 | 20.50 (-19.94, 60.94) 0.3204 |
| High | 479.86 (413.49, 546.22) <0.0001 | 99.81 (51.85, 147.77) <0.0001 | 79.32 (37.65, 120.99) 0.0002 |
| Baseline FEV 1 (mL) |  |  |  |
| Iron,refigerated (umol/L) | 24.89 (21.33, 28.46) <0.0001 | 6.63 (4.04, 9.21) <0.0001 | 5.80 (3.49, 8.11) <0.0001 |
| Iron,refigerated (umol/L) Tertile |  |  |  |
| Low | 0 | 0 | 0 |
| Medium | 178.00 (122.59, 233.40) <0.0001 | 26.93 (-12.47, 66.34) 0.1804 | 16.22 (-18.77, 51.21) 0.3636 |
| High | 388.10 (332.45, 443.75) <0.0001 | 97.22 (56.90, 137.54) <0.0001 | 82.40 (46.34, 118.45) <0.0001 |
| Baseline FEF 25-75% (mL/s) |  |  |  |
| Iron,refigerated (umol/L) | 20.80 (15.52, 26.08) <0.0001 | 6.60 (2.12, 11.08) 0.0039 | 6.72 (2.30, 11.13) 0.0029 |
| Iron,refigerated (umol/L) Tertile |  |  |  |
| Low | 0 | 0 | 0 |
| Medium | 100.26 (18.26, 182.26) 0.0166 | 7.25 (-61.06, 75.56) 0.8352 | 8.10 (-58.71, 74.92) 0.8121 |
| High | 325.25 (242.89, 407.60) <0.0001 | 89.45 (19.57, 159.34) 0.0121 | 89.96 (21.12, 158.81) 0.0105 |
| ***Stratification by food*** |  |  |  |
| Had food= Yes |  |  |  |
| Baseline FVC (mL) |  |  |  |
| Iron,refigerated (umol/L) | 40.32 (31.38, 49.25) <0.0001 | 13.86 (7.32, 20.40) <0.0001 | 11.27 (5.50, 17.04) 0.0001 |
| Iron,refigerated (umol/L) Tertile |  |  |  |
| Low | 0 | 0 | 0 |
| Medium | 348.00 (208.83, 487.17) <0.0001 | 122.15 (22.42, 221.87) 0.0165 | 85.20 (-1.46, 171.86) 0.0542 |
| High | 593.09 (455.95, 730.23) <0.0001 | 171.94 (71.43, 272.46) 0.0008 | 127.81 (39.41, 216.20) 0.0047 |
| Baseline FEV 1 (mL) |  |  |  |
| Iron,refigerated (umol/L) | 31.33 (23.88, 38.77) <0.0001 | 12.31 (6.96, 17.66) <0.0001 | 10.01 (5.15, 14.88) <0.0001 |
| Iron,refigerated (umol/L) Tertile |  |  |  |
| Low | 0 | 0 | 0 |
| Medium | 246.31 (130.29, 362.34) <0.0001 | 99.64 (17.99, 181.29) 0.0169 | 71.90 (-1.18, 144.98) 0.0540 |
| High | 453.98 (339.65, 568.31) <0.0001 | 148.15 (65.86, 230.44) 0.0004 | 112.18 (37.63, 186.73) 0.0032 |
| Baseline FEF 25-75% (mL/s) |  |  |  |
| Iron,refigerated (umol/L) | 21.52 (10.47, 32.57) 0.0001 | 8.82 (-0.37, 18.01) 0.0601 | 7.24 (-1.96, 16.45) 0.1231 |
| Iron,refigerated (umol/L) Tertile |  |  |  |
| Low | 0 | 0 | 0 |
| Medium | 120.02 (-51.91, 291.95) 0.1715 | 59.48 (-80.52, 199.49) 0.4051 | 41.93 (-96.05, 179.90) 0.5516 |
| High | 288.61 (119.19, 458.03) 0.0009 | 78.34 (-62.76, 219.45) 0.2767 | 58.06 (-82.68, 198.79) 0.4189 |
| Had food= No |  |  |  |
| Baseline FVC (mL) |  |  |  |
| Iron,refigerated (umol/L) | 34.06 (29.02, 39.10) <0.0001 | 5.54 (1.92, 9.16) 0.0027 | 3.93 (0.85, 7.01) 0.0124 |
| Iron,refigerated (umol/L) Tertile |  |  |  |
| Low | 0 | 0 | 0 |
| Medium | 260.92 (181.75, 340.10) <0.0001 | 21.05 (-34.52, 76.62) 0.4578 | -2.03 (-48.81, 44.75) 0.9322 |
| High | 532.19 (452.97, 611.41) <0.0001 | 96.42 (39.61, 153.23) 0.0009 | 65.96 (17.74, 114.18) 0.0074 |
| Baseline FEV 1 (mL) |  |  |  |
| Iron,refigerated (umol/L) | 27.32 (23.09, 31.55) <0.0001 | 5.66 (2.65, 8.68) 0.0002 | 4.76 (2.08, 7.44) 0.0005 |
| Iron,refigerated (umol/L) Tertile |  |  |  |
| Low | 0 | 0 | 0 |
| Medium | 177.71 (111.26, 244.16) <0.0001 | 8.86 (-37.38, 55.11) 0.7071 | -5.10 (-45.82, 35.62) 0.8061 |
| High | 428.16 (361.67, 494.65) <0.0001 | 91.27 (43.99, 138.55) 0.0002 | 72.80 (30.82, 114.77) 0.0007 |
| Baseline FEF 25-75% (mL/s) |  |  |  |
| Iron,refigerated (umol/L) | 23.22 (16.91, 29.53) <0.0001 | 6.26 (1.05, 11.46) 0.0185 | 6.84 (1.71, 11.97) 0.0090 |
| Iron,refigerated (umol/L) Tertile |  |  |  |
| Low | 0 | 0 | 0 |
| Medium | 87.66 (-11.32, 186.65) 0.0827 | -12.12 (-92.00, 67.77) 0.7663 | -11.92 (-89.93, 66.09) 0.7646 |
| High | 369.36 (270.32, 468.40) <0.0001 | 90.92 (9.25, 172.60) 0.0292 | 95.38 (14.97, 175.79) 0.0201 |
| Had food= Unknown |  |  |  |
| Baseline FVC (mL) |  |  |  |
| Iron,refigerated (umol/L) | 17.42 (-9.75, 44.59) 0.2110 | 5.83 (-13.07, 24.74) 0.5466 |  |
| Iron,refigerated (umol/L) Tertile |  |  |  |
| Low | 0 | 0 |  |
| Medium | 191.06 (-187.14, 569.26) 0.3239 | 122.85 (-142.05, 387.75) 0.3651 |  |
| High | 291.72 (-141.56, 725.00) 0.1892 | 97.05 (-203.97, 398.07) 0.5286 |  |
| Baseline FEV 1 (mL) |  |  |  |
| Iron,refigerated (umol/L) | 11.08 (-12.66, 34.82) 0.3619 | 1.32 (-15.22, 17.87) 0.8759 |  |
| Iron,refigerated (umol/L) Tertile |  |  |  |
| Low | 0 | 0 |  |
| Medium | 115.79 (-214.43, 446.01) 0.4931 | 64.28 (-167.68, 296.24) 0.5880 |  |
| High | 228.44 (-149.87, 606.76) 0.2387 | 77.12 (-186.47, 340.70) 0.5674 |  |
| Baseline FEF 25-75% (mL/s) |  |  |  |
| Iron,refigerated (umol/L) | 2.06 (-33.59, 37.71) 0.9101 | -6.82 (-36.45, 22.81) 0.6527 |  |
| Iron,refigerated (umol/L) Tertile |  |  |  |
| Low | 0 | 0 |  |
| Medium | 38.04 (-458.43, 534.50) 0.8809 | 5.16 (-411.20, 421.53) 0.9806 |  |
| High | 160.67 (-408.10, 729.44) 0.5807 | 37.56 (-435.57, 510.70) 0.8766 |  |
| Total |  |  |  |
| Baseline FVC (mL) |  |  |  |
| Iron,refigerated (umol/L) | 35.14 (30.80, 39.47) <0.0001 | 7.54 (4.41, 10.66) <0.0001 | 5.45 (2.78, 8.13) <0.0001 |
| Iron,refigerated (umol/L) Tertile |  |  |  |
| Low | 0 | 0 | 0 |
| Medium | 279.51 (211.79, 347.22) <0.0001 | 46.08 (-1.63, 93.79) 0.0584 | 20.50 (-19.94, 60.94) 0.3204 |
| High | 541.68 (473.96, 609.40) <0.0001 | 114.73 (65.98, 163.47) <0.0001 | 79.32 (37.65, 120.99) 0.0002 |
| Baseline FEV 1 (mL) |  |  |  |
| Iron,refigerated (umol/L) | 27.88 (24.24, 31.51) <0.0001 | 7.09 (4.50, 9.68) <0.0001 | 5.80 (3.49, 8.11) <0.0001 |
| Iron,refigerated (umol/L) Tertile |  |  |  |
| Low | 0 | 0 | 0 |
| Medium | 192.06 (135.27, 248.86) <0.0001 | 31.07 (-8.51, 70.66) 0.1240 | 16.22 (-18.77, 51.21) 0.3636 |
| High | 429.98 (373.18, 486.78) <0.0001 | 104.49 (64.05, 144.94) <0.0001 | 82.40 (46.34, 118.45) <0.0001 |
| Baseline FEF 25-75% (mL/s) |  |  |  |
| Iron,refigerated (umol/L) | 22.33 (16.92, 27.75) <0.0001 | 6.42 (1.95, 10.89) 0.0049 | 6.72 (2.30, 11.13) 0.0029 |
| Iron,refigerated (umol/L) Tertile |  |  |  |
| Low | 0 | 0 | 0 |
| Medium | 93.47 (8.95, 177.98) 0.0302 | 5.72 (-62.58, 74.03) 0.8695 | 8.10 (-58.71, 74.92) 0.8121 |
| High | 345.06 (260.54, 429.59) <0.0001 | 86.59 (16.80, 156.37) 0.0151 | 89.96 (21.12, 158.81) 0.0105 |
| ***Stratification by diabetes*** |  |  |  |
| Diabetes= Yes |  |  |  |
| Baseline FVC (mL) |  |  |  |
| Iron,refigerated (umol/L) | 59.55 (42.28, 76.82) <0.0001 | 16.67 (3.88, 29.46) 0.0110 | 15.66 (3.92, 27.39) 0.0093 |
| Iron,refigerated (umol/L) Tertile |  |  |  |
| Low | 0 | 0 | 0 |
| Medium | 379.64 (154.56, 604.73) 0.0010 | 112.21 (-47.17, 271.59) 0.1684 | 149.64 (2.75, 296.54) 0.0466 |
| High | 828.36 (590.84, 1065.89) <0.0001 | 241.56 (65.85, 417.28) 0.0074 | 221.84 (60.21, 383.47) 0.0075 |
| Baseline FEV 1 (mL) |  |  |  |
| Iron,refigerated (umol/L) | 44.54 (30.41, 58.67) <0.0001 | 12.46 (1.83, 23.08) 0.0221 | 11.73 (1.62, 21.85) 0.0236 |
| Iron,refigerated (umol/L) Tertile |  |  |  |
| Low | 0 | 0 | 0 |
| Medium | 280.71 (96.60, 464.81) 0.0030 | 85.90 (-46.56, 218.36) 0.2045 | 121.24 (-5.37, 247.85) 0.0614 |
| High | 623.73 (429.45, 818.01) <0.0001 | 176.41 (30.37, 322.45) 0.0184 | 165.45 (26.13, 304.76) 0.0205 |
| Baseline FEF 25-75% (mL/s) |  |  |  |
| Iron,refigerated (umol/L) | 30.10 (9.32, 50.88) 0.0048 | 6.20 (-11.67, 24.07) 0.4971 | 4.96 (-13.45, 23.36) 0.5980 |
| Iron,refigerated (umol/L) Tertile |  |  |  |
| Low | 0 | 0 | 0 |
| Medium | 146.86 (-124.29, 418.01) 0.2891 | 15.18 (-207.79, 238.14) 0.8939 | 45.25 (-185.51, 276.00) 0.7010 |
| High | 423.58 (137.44, 709.72) 0.0039 | 63.26 (-182.56, 309.09) 0.6143 | 42.31 (-211.59, 296.21) 0.7442 |
| Diabetes= No |  |  |  |
| Baseline FVC (mL) |  |  |  |
| Iron,refigerated (umol/L) | 32.38 (27.92, 36.84) <0.0001 | 6.58 (3.35, 9.81) <0.0001 | 5.16 (2.40, 7.92) 0.0002 |
| Iron,refigerated (umol/L) Tertile |  |  |  |
| Low | 0 | 0 | 0 |
| Medium | 261.69 (190.96, 332.42) <0.0001 | 41.84 (-8.39, 92.07) 0.1026 | 20.35 (-22.13, 62.83) 0.3478 |
| High | 504.15 (433.70, 574.60) <0.0001 | 100.28 (49.27, 151.29) 0.0001 | 74.71 (31.19, 118.22) 0.0008 |
| Baseline FEV 1 (mL) |  |  |  |
| Iron,refigerated (umol/L) | 25.70 (21.97, 29.43) <0.0001 | 6.59 (3.90, 9.28) <0.0001 | 5.72 (3.32, 8.11) <0.0001 |
| Iron,refigerated (umol/L) Tertile |  |  |  |
| Low | 0 | 0 | 0 |
| Medium | 176.00 (116.80, 235.21) <0.0001 | 29.29 (-12.53, 71.10) 0.1699 | 16.45 (-20.38, 53.28) 0.3814 |
| High | 400.96 (341.99, 459.92) <0.0001 | 97.73 (55.27, 140.19) <0.0001 | 81.63 (43.90, 119.36) <0.0001 |
| Baseline FEF 25-75% (mL/s) |  |  |  |
| Iron,refigerated (umol/L) | 20.47 (14.88, 26.06) <0.0001 | 6.59 (1.93, 11.25) 0.0056 | 7.00 (2.41, 11.59) 0.0028 |
| Iron,refigerated (umol/L) Tertile |  |  |  |
| Low | 0 | 0 | 0 |
| Medium | 80.62 (-8.00, 169.24) 0.0746 | 14.16 (-58.32, 86.64) 0.7018 | 15.85 (-54.84, 86.55) 0.6603 |
| High | 323.06 (234.79, 411.32) <0.0001 | 93.56 (19.96, 167.17) 0.0128 | 98.70 (26.27, 171.12) 0.0076 |
| Diabetes= Unknown |  |  |  |
| Baseline FVC (mL) |  |  |  |
| Iron,refigerated (umol/L) | 27.63 (-11.37, 66.64) 0.1688 | 4.67 (-28.96, 38.29) 0.7864 | -8.76 (-47.91, 30.40) 0.6628 |
| Iron,refigerated (umol/L) Tertile |  |  |  |
| Low | 0 | 0 | 0 |
| Medium | -88.97 (-569.77, 391.83) 0.7178 | -221.73 (-622.57, 179.11) 0.2818 | -39.70 (-455.58, 376.17) 0.8523 |
| High | 290.26 (-207.75, 788.27) 0.2567 | 89.25 (-323.97, 502.47) 0.6733 | 55.88 (-410.54, 522.30) 0.8152 |
| Baseline FEV 1 (mL) |  |  |  |
| Iron,refigerated (umol/L) | 7.61 (-23.14, 38.36) 0.6290 | -1.58 (-27.87, 24.71) 0.9064 | -17.41 (-48.43, 13.61) 0.2762 |
| Iron,refigerated (umol/L) Tertile |  |  |  |
| Low | 0 | 0 | 0 |
| Medium | -166.01 (-543.92, 211.90) 0.3918 | -218.64 (-532.14, 94.86) 0.1758 | -101.44 (-433.29,230.41) 0.5516 |
| High | 43.07 (-348.37, 434.51) 0.8298 | -10.17 (-333.35, 313.00) 0.9510 | -93.34 (-465.53, 278.84) 0.6250 |
| Baseline FEF 25-75% (mL/s) |  |  |  |
| Iron,refigerated (umol/L) | -22.79 (-69.58, 24.01) 0.3427 | -7.88 (-50.05, 34.30) 0.7154 | -49.38 (-101.13, 2.36) 0.0667 |
| Iron,refigerated (umol/L) Tertile |  |  |  |
| Low | 0 | 0 | 0 |
| Medium | -433.84 (-1006.83, 139.14) 0.1417 | -382.13 (-886.52, 122.25) 0.1419 | -397.47 (-947.41,152.47) 0.1623 |
| High | -390.69 (-984.18, 202.81) 0.2007 | -173.72 (-693.68, 346.24) 0.5146 | -541.41 (-1158.19, 75.36) 0.0911 |
| Total |  |  |  |
| Baseline FVC (mL) |  |  |  |
| Iron,refigerated (umol/L) | 33.82 (29.53, 38.10) <0.0001 | 7.19 (4.07, 10.31) <0.0001 | 5.45 (2.78, 8.13) <0.0001 |
| Iron,refigerated (umol/L) Tertile |  |  |  |
| Low | 0 | 0 | 0 |
| Medium | 266.74 (199.81, 333.67) <0.0001 | 40.98 (-6.65, 88.61) 0.0918 | 20.50 (-19.94, 60.94) 0.3204 |
| High | 523.56 (456.60, 590.52) <0.0001 | 110.42 (61.77, 159.08) <0.0001 | 79.32 (37.65, 120.99) 0.0002 |
| Baseline FEV 1 (mL) |  |  |  |
| Iron,refigerated (umol/L) | 26.55 (22.97, 30.13) <0.0001 | 6.88 (4.29, 9.47) <0.0001 | 5.80 (3.49, 8.11) <0.0001 |
| Iron,refigerated (umol/L) Tertile |  |  |  |
| Low | 0 | 0 | 0 |
| Medium | 179.71 (123.81, 235.61) <0.0001 | 28.02 (-11.55, 67.60) 0.1652 | 16.22 (-18.77, 51.21) 0.3636 |
| High | 411.37 (355.44, 467.29) <0.0001 | 101.84 (61.42, 142.27) <0.0001 | 82.40 (46.34, 118.45) <0.0001 |
| Baseline FEF 25-75% (mL/s) |  |  |  |
| Iron,refigerated (umol/L) | 20.56 (15.21, 25.92) <0.0001 | 6.41 (1.93, 10.88) 0.0050 | 6.72 (2.30, 11.13) 0.0029 |
| Iron,refigerated (umol/L) Tertile |  |  |  |
| Low | 0 | 0 | 0 |
| Medium | 78.09 (-5.49, 161.68) 0.0671 | 5.84 (-62.51, 74.19) 0.8669 | 8.10 (-58.71, 74.92) 0.8121 |
| High | 319.62 (236.00, 403.25) <0.0001 | 86.30 (16.48, 156.12) 0.0154 | 89.96 (21.12, 158.81) 0.0105 |
| ***Stratification by cholesterol*** | ***Stratification by*** | ***Stratification by*** | ***Stratification by*** |
| Cholesterol (mmol/L) Tertile= Low |  |  |  |
| Baseline FVC (mL) |  |  |  |
| Iron,refigerated (umol/L) | 27.62 (20.29, 34.94) <0.0001 | 4.61 (-0.92, 10.13) 0.1024 | 4.48 (-0.04, 8.99) 0.0520 |
| Iron,refigerated (umol/L) Tertile |  |  |  |
| Low | 0 | 0 | 0 |
| Medium | 231.74 (115.07, 348.42) 0.0001 | -12.39 (-99.23, 74.45) 0.7798 | -18.58 (-89.36, 52.19) 0.6069 |
| High | 452.36 (335.98, 568.75) <0.0001 | 80.06 (-7.94, 168.06) 0.0747 | 80.91 (8.99, 152.84) 0.0276 |
| Baseline FEV 1 (mL) |  |  |  |
| Iron,refigerated (umol/L) | 24.00 (17.77, 30.24) <0.0001 | 5.59 (0.94, 10.23) 0.0185 | 4.96 (0.95, 8.97) 0.0155 |
| Iron,refigerated (umol/L) Tertile |  |  |  |
| Low | 0 | 0 | 0 |
| Medium | 185.64 (86.33, 284.94) 0.0003 | -5.83 (-78.87, 67.21) 0.8757 | -16.28 (-79.16, 46.61) 0.6120 |
| High | 390.20 (291.14, 489.25) <0.0001 | 82.22 (8.21, 156.23) 0.0296 | 72.84 (8.93, 136.75) 0.0256 |
| Baseline FEF 25-75% (mL/s) |  |  |  |
| Iron,refigerated (umol/L) | 21.60 (12.34, 30.86) <0.0001 | 6.02 (-1.77, 13.80) 0.1299 | 4.88 (-2.75, 12.51) 0.2102 |
| Iron,refigerated (umol/L) Tertile |  |  |  |
| Low | 0 | 0 | 0 |
| Medium | 115.44 (-32.13, 263.00) 0.1254 | -39.23 (-161.63, 83.17) 0.5300 | -51.68 (-171.40, 68.03) 0.3976 |
| High | 345.78 (198.58, 492.97) <0.0001 | 61.53 (-62.50, 185.56) 0.3310 | 43.03 (-78.64, 164.69) 0.4883 |
| Cholesterol (mmol/L) Tertile= Medium |  |  |  |
| Baseline FVC (mL) |  |  |  |
| Iron,refigerated (umol/L) | 40.14 (32.72, 47.55) <0.0001 | 7.64 (2.19, 13.09) 0.0061 | 5.62 (0.85, 10.38) 0.0210 |
| Iron,refigerated (umol/L) Tertile |  |  |  |
| Low | 0 | 0 | 0 |
| Medium | 310.11 (195.71, 424.51) <0.0001 | 50.63 (-30.79, 132.05) 0.2231 | 29.95 (-40.24, 100.14) 0.4031 |
| High | 595.06 (479.33, 710.79) <0.0001 | 104.68 (20.17, 189.19) 0.0153 | 80.90 (7.26, 154.53) 0.0314 |
| Baseline FEV 1 (mL) |  |  |  |
| Iron,refigerated (umol/L) | 33.43 (27.22, 39.64) <0.0001 | 8.31 (3.81, 12.81) 0.0003 | 7.01 (2.92, 11.09) 0.0008 |
| Iron,refigerated (umol/L) Tertile |  |  |  |
| Low | 0 | 0 | 0 |
| Medium | 216.81 (120.93, 312.70) <0.0001 | 33.65 (-33.62, 100.91) 0.3270 | 21.64 (-38.54, 81.82) 0.4810 |
| High | 492.95 (395.95, 589.94) <0.0001 | 109.87 (40.06, 179.69) 0.0021 | 96.07 (32.94, 159.20) 0.0029 |
| Baseline FEF 25-75% (mL/s) |  |  |  |
| Iron,refigerated (umol/L) | 30.87 (21.52, 40.23) <0.0001 | 10.11 (2.30, 17.91) 0.0112 | 10.88 (3.17, 18.60) 0.0058 |
| Iron,refigerated (umol/L) Tertile |  |  |  |
| Low | 0 | 0 | 0 |
| Medium | 92.66 (-51.34, 236.65) 0.2074 | -19.07 (-135.66, 97.52) 0.7486 | -15.95 (-129.60, 97.70) 0.7833 |
| High | 441.34 (295.67, 587.00) <0.0001 | 113.39 (-7.63, 234.40) 0.0665 | 127.37 (8.15, 246.60) 0.0364 |
| Cholesterol (mmol/L) Tertile= High |  |  |  |
| Baseline FVC (mL) |  |  |  |
| Iron,refigerated (umol/L) | 40.17 (32.36, 47.98) <0.0001 | 8.51 (3.23, 13.79) 0.0016 | 6.21 (1.55, 10.86) 0.0090 |
| Iron,refigerated (umol/L) Tertile |  |  |  |
| Low | 0 | 0 | 0 |
| Medium | 331.71 (209.86, 453.57) <0.0001 | 83.61 (3.61, 163.61) 0.0407 | 56.02 (-14.03, 126.07) 0.1172 |
| High | 618.52 (497.65, 739.40) <0.0001 | 133.30 (51.52, 215.09) 0.0014 | 80.97 (9.08, 152.85) 0.0274 |
| Baseline FEV 1 (mL) |  |  |  |
| Iron,refigerated (umol/L) | 29.99 (23.65, 36.34) <0.0001 | 6.52 (2.16, 10.89) 0.0034 | 5.34 (1.39, 9.29) 0.0082 |
| Iron,refigerated (umol/L) Tertile |  |  |  |
| Low | 0 | 0 | 0 |
| Medium | 230.92 (132.07, 329.76) <0.0001 | 59.18 (-6.87, 125.24) 0.0792 | 44.15 (-15.32, 103.63) 0.1458 |
| High | 470.89 (372.84, 568.95) <0.0001 | 112.00 (44.47, 179.52) 0.0012 | 79.71 (18.68, 140.75) 0.0105 |
| Baseline FEF 25-75% (mL/s) |  |  |  |
| Iron,refigerated (umol/L) | 21.46 (12.33, 30.59) <0.0001 | 3.27 (-4.47, 11.00) 0.4082 | 4.12 (-3.53, 11.77) 0.2912 |
| Iron,refigerated (umol/L) Tertile |  |  |  |
| Low | 0 | 0 | 0 |
| Medium | 184.46 (42.22, 326.69) 0.0111 | 82.89 (-34.30, 200.08) 0.1658 | 88.85 (-26.18, 203.88) 0.1302 |
| High | 373.18 (232.09, 514.28) <0.0001 | 99.24 (-20.56, 219.04) 0.1046 | 100.53 (-17.52, 218.57) 0.0953 |
| Total |  |  |  |
| Baseline FVC (mL) |  |  |  |
| Iron,refigerated (umol/L) | 35.81 (31.47, 40.15) <0.0001 | 7.04 (3.91, 10.17) <0.0001 | 5.45 (2.78, 8.13) <0.0001 |
| Iron,refigerated (umol/L) Tertile |  |  |  |
| Low | 0 | 0 | 0 |
| Medium | 288.36 (220.53, 356.18) <0.0001 | 39.11 (-8.65, 86.88) 0.1086 | 20.50 (-19.94, 60.94) 0.3204 |
| High | 554.38 (486.54, 622.22) <0.0001 | 107.20 (58.33, 156.08) <0.0001 | 79.32 (37.65, 120.99) 0.0002 |
| Baseline FEV 1 (mL) |  |  |  |
| Iron,refigerated (umol/L) | 29.06 (25.45, 32.68) <0.0001 | 6.99 (4.39, 9.59) <0.0001 | 5.80 (3.49, 8.11) <0.0001 |
| Iron,refigerated (umol/L) Tertile |  |  |  |
| Low | 0 | 0 | 0 |
| Medium | 209.80 (153.31, 266.29) <0.0001 | 29.47 (-10.22, 69.16) 0.1457 | 16.22 (-18.77, 51.21) 0.3636 |
| High | 450.90 (394.40, 507.40) <0.0001 | 103.18 (62.57, 143.79) <0.0001 | 82.40 (46.34, 118.45) <0.0001 |
| Baseline FEF 25-75% (mL/s) |  |  |  |
| Iron,refigerated (umol/L) | 24.65 (19.32, 29.99) <0.0001 | 6.77 (2.28, 11.25) 0.0031 | 6.72 (2.30, 11.13) 0.0029 |
| Iron,refigerated (umol/L) Tertile |  |  |  |
| Low | 0 | 0 | 0 |
| Medium | 130.32 (46.95, 213.69) 0.0022 | 10.57 (-57.87, 79.00) 0.7622 | 8.10 (-58.71, 74.92) 0.8121 |
| High | 384.74 (301.34, 468.13) <0.0001 | 92.45 (22.42, 162.47) 0.0097 | 89.96 (21.12, 158.81) 0.0105 |

**Note**; **β (95%CI) Pvalue;β**;Beta value ;**CI** ;95% confidence interval，P; P-value**.Exposure variable**: serum iron(umol/L). Non-adjusted model adjust for: None.Adjust I model adjust for: Age; Gender; Race/Hispanic origin. Adjust II model adjust for: Age; Gender; Race/Hispanic origin; Education level; Thoracic/abdominal surgery; Respiratory disease; Cigarette; Heart attack; Stroke; Body Mass Index; Systolic Blood Pressure; Diastolic Blood Pressure; Standing Height (cm).*** When covariates are used as analyzed variables, the adjustment variables are not added to this covariate adjustment Outcome variable**: baseline FVC (mL); baseline FEV 1 (mL)；Baseline FEF 25-75% (mL/s). This table was generated using EasyTok statistical software (www.empowerstats. com) and R software.
