# Supplementary figures and images for: Correlation between serum iron levels and pulmonary function: A cross-sectional analysis based on NHANES database 5319 cases
Source: Medicine (Baltimore). 2023 Dec 15;102(50):e36449. doi: 10.1097/MD.0000000000036449 (PMC10727669; doi:10.1097/MD.0000000000036449)

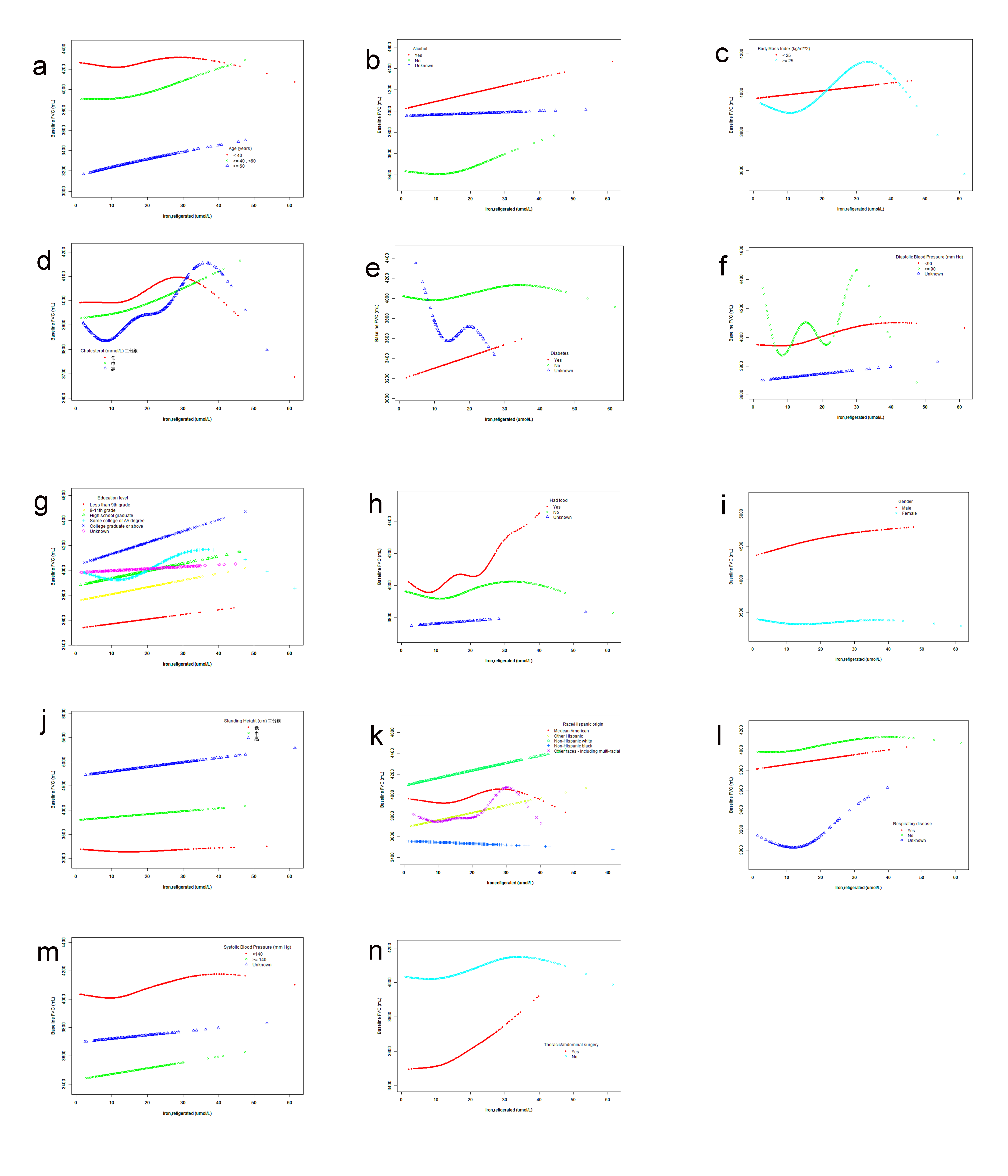

Supplement: Supplementary file 2 [file medi-102-e36449-s002.tif]

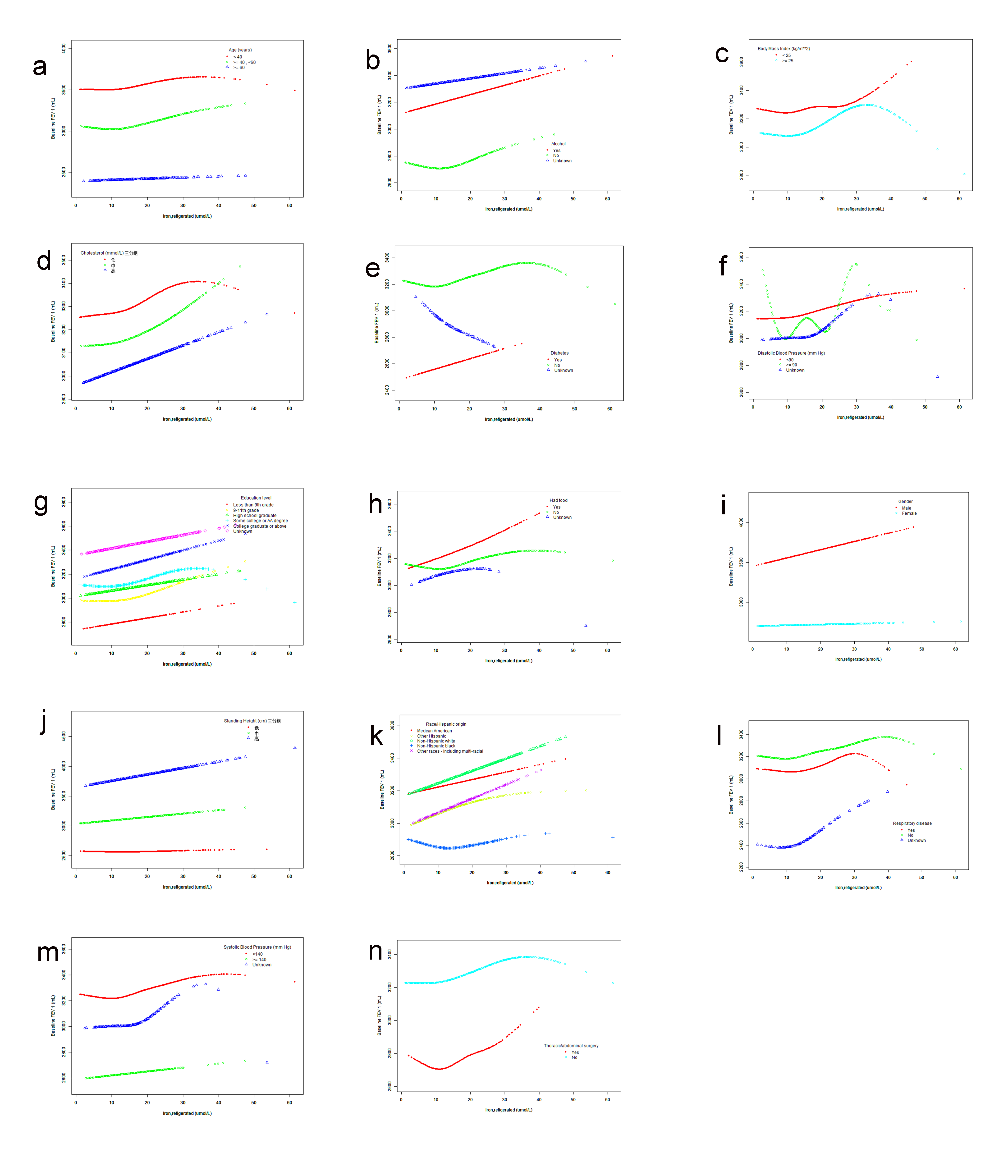

Supplement: Supplementary file 3 [file medi-102-e36449-s003.tif]

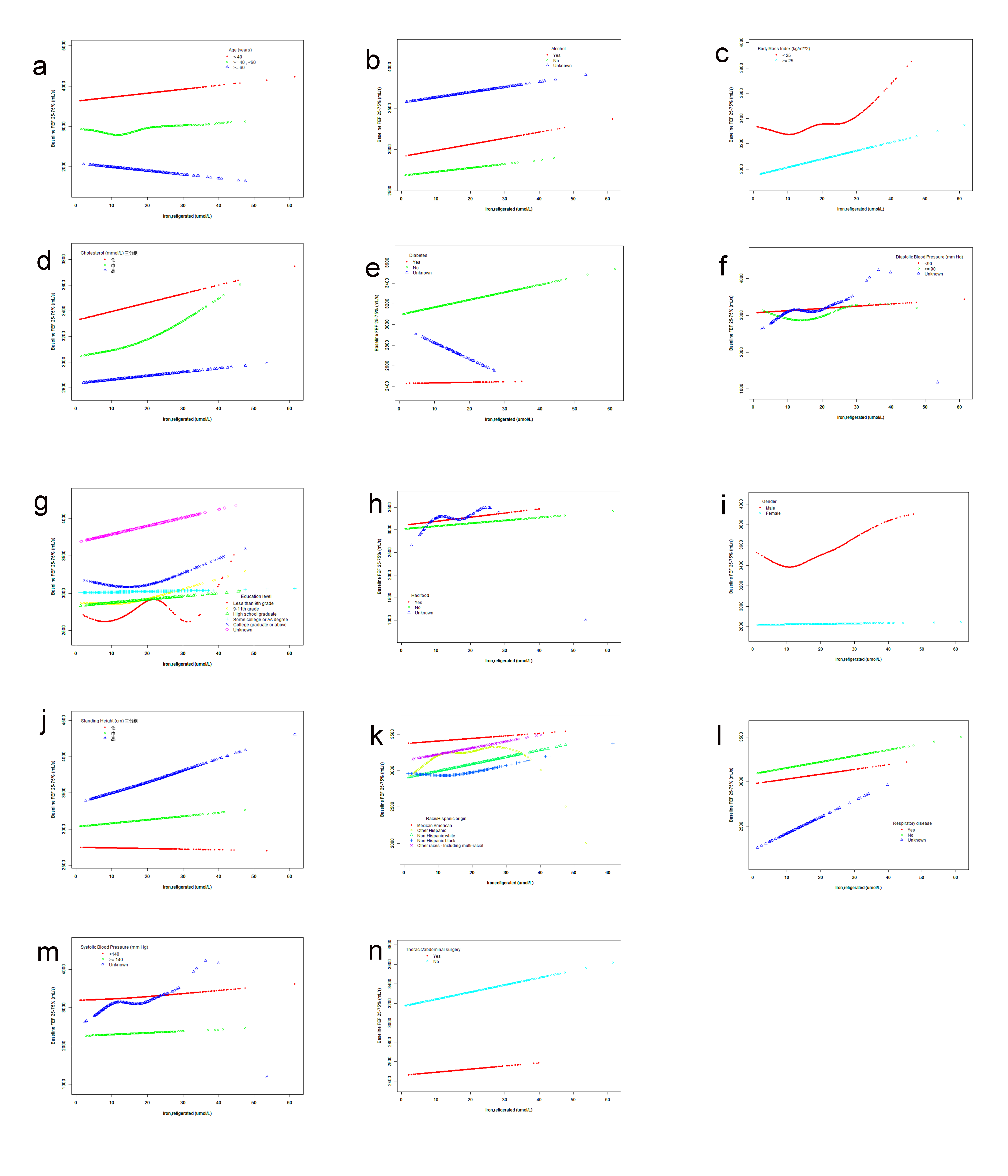

Supplement: Supplementary file 4 [file medi-102-e36449-s004.tif]
